# SUMO2/3 modification of transcription-associated proteins controls cell viability in response to oxygen and glucose deprivation-mediated stress

**DOI:** 10.1038/s41420-025-02513-w

**Published:** 2025-05-10

**Authors:** Francisco Gallardo-Chamizo, Román González-Prieto, Vahid Jafari, Noelia Luna-Peláez, Alfred C. O. Vertegaal, Mario García-Domínguez

**Affiliations:** 1 Andalusian Centre for Molecular Biology and Regenerative Medicine-CABIMER, CSIC-Universidad de Sevilla-Universidad Pablo de Olavide, Seville, Spain; 2 Department of Cell and Chemical Biology, Leiden University Medical Center, Leiden, Netherlands

**Keywords:** Proteomics, Cell death, Cell signalling

## Abstract

Because limited oxygen and glucose supply to tissues is a serious challenge that cells must properly measure to decide between surviving or triggering cell death, organisms have developed accurate mechanisms for sensing and signaling these conditions. In recent years, signaling through posttranslational modification of proteins by covalent attachment of the Small Ubiquitin-like Modifier (SUMO) is gaining notoriety. Enhanced sumoylation in response to oxygen and glucose deprivation (OGD) constitutes a safeguard mechanism for cells and a new avenue for therapeutic intervention. However, indiscriminate global sumoylation can limit the therapeutic potential that a more precise action on selected targets would have. To clear up this, we have conducted a proteomic approach in P19 cells to identify specific SUMO targets responding to OGD and to investigate the potential that these targets and their sumoylation have in preserving cells from death. Proteins undergoing sumoylation in response to OGD are mostly related to transcription and RNA processing, and the majority of them are rapidly desumoylated when restoring oxygen and glucose (ROG), confirming the high dynamics of this modification. Since OGD is linked to brain ischemia, we have also studied cells differentiated into neurons. However, no major differences have been observed between the SUMO-proteomes of proliferating and differentiated cells. We show that the overexpression of the transcription factor SOX2 or the SUMO ligase PIAS4 has a manifest cell protective effect largely depending on their sumoylation, and that maintaining the sumoylation capacity of the coregulator NAB2 is also important to face OGD. Conversely, sumoylation of the pluripotency factor OCT4, which is sumoylated under OGD, and is a target of the SUMO protease SENP7 for desumoylation after ROG, seems to block its cell survival-promoting capacity. Thus, better outcomes in cell protection would rely on the appropriate combination of sumoylated and non-sumoylated forms of selected factors.

## Introduction

Limited physiological or pathological supply of oxygen and glucose to cells seriously compromises cell viability. Many cell types in the organism and cancer cells, especially those inside solid tumors, successfully adapt to these conditions under a mild limiting supply. However, severe oxygen and glucose deprivation (OGD) has dramatic consequences for cell survival. For instance, ischemia, especially in the brain, depending on the duration of the process and the affected surface, can cause drastic damage and a severe loss of functionality in the patient’s brain, profoundly affecting normal daily tasks, and constituting a serious public health problem nowadays.

Sumoylation of proteins has been reported to play a key role in promoting cell survival under OGD conditions. Sumoylation consists of the attachment of the Ubiquitin-like small polypeptide SUMO (Small Ubiquitin-like MOdifier) to Lys (K) residues, often embedded in the consensus sequence ΨKXE (Ψ, large hydrophobic residue; X, any residue), within proteins, as a posttranslational modification [1]. After proteolytic maturation by specific SUMO proteases of several C-terminal amino acids, SUMO is activated by the heterodimeric SAE1/UBA2 E1 enzyme to be transferred to the SUMO E2 conjugating enzyme UBC9, which sumoylates target proteins, frequently helped by an E3 ligase. The SUMO proteases are also involved in recycling SUMO from targets and the most studied are those of the SENP family [2]. SUMO ligases, through different mechanisms, facilitate SUMO transfer from UBC9 to target proteins, and those of the PIAS family have been extensively investigated [3]. Proteases and ligases are key SUMO regulators, which appear highly dysregulated in cancer [4].

SUMO is essential in vertebrates and participates in many physiological processes; thus, it also associates with multiple diseases [5]. Up to five SUMO molecules have been identified in humans. However, clear evidence of conjugation capacity and wide roles have been restricted to SUMO1, 2, and 3. SUMO2 and 3 are highly homologous and undistinguishable by antibodies and functionally, and as such they are usually referred to as SUMO2/3. Indeed, it is SUMO2/3, the SUMO paralog mostly associated with the response to OGD conditions. In contrast to SUMO1, SUMO2/3 is abundant in the cell in the unconjugated form and rapidly attached to proteins in response to several stress stimuli [6], including ischemia [7, 8]. Another difference between SUMO1 and SUMO2/3 is the presence in SUMO2/3 of a sumoylation consensus site, which facilitates the formation of poly-SUMO chains.

First under physiological conditions during hibernating torpor in squirrels and subsequently under pathological conditions by inducing ischemia in mice by middle cerebral artery occlusion, increased and massive sumoylation of proteins has been correlated with neuroprotection (reviewed in [9]). Indeed, diverse reports have established better outcomes in cell viability associated with SUMO or UBC9 overexpression and worse outcomes associated with SUMO silencing or SENP proteases overexpression [10–14]. However, these approaches surely lead to indiscriminate sumoylation of many proteins, which can limit the positive outcomes due to the negative effects of certain modified proteins. Thus, the identification of specific targets and the precise characterization of their roles in the sumoylated form is fundamental to more precisely and selectively optimize intervention at this level. In this context, a few works have explored the identification of SUMO targets in response to OGD, but with limited outcomes [15, 16]. In one case, a reduced number of proteins have been identified, and in general, these studies lack functional analysis highlighting the relevance of target modification in the context of cell viability under OGD-mediated stress.

In this study, we have investigated the changes in the SUMO2 proteome of cells subjected to OGD, taking advantage of the differentiable P19 cell line, to compare SUMO2 targets under normal growth conditions with those in differentiated cells. Our results indicate that dozens of proteins are similarly modified under both conditions, mainly grouping into transcription-related functional categories. In addition, we also show how overexpression of sumoylation mutants of selected factors may lead to different effects on cell viability. This opens the possibility of therapeutically interfering with the sumoylation of specific factors to get better outcomes in cell viability in response to ischemia.

## Results

### Proteomic analysis reveals SUMO2-modification of multiple transcription-associated proteins in response to oxygen and glucose deprivation

As the sumoylation of proteins has been associated with tolerance to stress conditions, and OGD has serious consequences under pathological and physiological circumstances, we aimed at identifying proteins modified by SUMO in response to OGD to shed light on mechanisms triggered in response to deleterious conditions and to discover new targets for putative therapeutic intervention. For this purpose, we have performed a proteomic approach to purify and identify SUMO2 conjugates. For the study, we have chosen mouse embryonal carcinoma P19 cells as they have the interest of presenting pluripotent characteristics, being easily differentiable through several well-established protocols [17, 18].

**Fig. 1:**
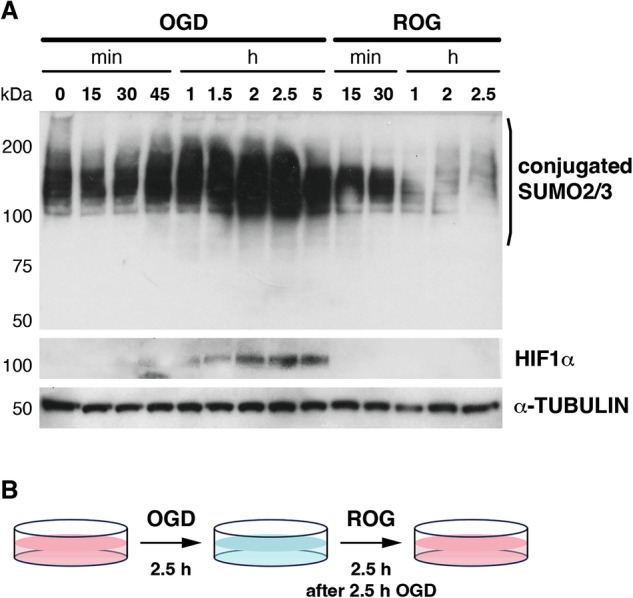
Dynamics of protein sumoylation by SUMO2/3 under OGD and after ROG. **A** P19 cells were subjected to the indicated times of OGD and to the indicated times of ROG after 2.5 h of OGD, and cell lysates under denaturing conditions were probed by western blot with anti SUMO2/3 antibodies. Induction of HIF1α was also registered as a control of OGD-associated hypoxia. α-TUBULIN was revealed as a loading control. Twenty micrograms of total protein were loaded per lane. Three independent experiments were conducted and representative western blots are shown. **B** Schematic representation of the workflow for OGD and ROG times in the next experiments.

We first determined the appropriate timing in P19 cells for the increase in sumoylation under OGD and the decrease after restoring oxygen and glucose (ROG). Kinetic analysis indicated that maximal modification was reached after 2.5 h of OGD and that 2.5 h of ROG was enough to restore normal sumoylation levels (Fig. 1A). Changes in the level of the hypoxia factor HIF1α were recorded as a control of the oxygen deprivation process (Fig. 1A). Thus, for proteomic studies, we established a protocol of 2.5 h of OGD and 2.5 h of ROG after 2.5 h of OGD (Fig. 1B). To facilitate purification of sumoylated proteins, stable cell lines expressing a 10× His-tagged (His_10_) SUMO2 construct [19] were generated. Several antibiotic-resistant clones were isolated and tested for expression of His_10_-SUMO2 using anti-His antibodies (Supplementary Fig. S1A). We selected clone 5 (designated as #A5 cells from now on) for further experiments as it presented similar levels of expression of endogenous SUMO2/3 and His_10_-SUMO2, as determined by western blot with anti-SUMO2/3 antibodies, which revealed a double band corresponding to the endogenous protein and the His_10_-tagged transgenic version with lower migration (Supplementary Fig. S1B).

**Fig. 2:**
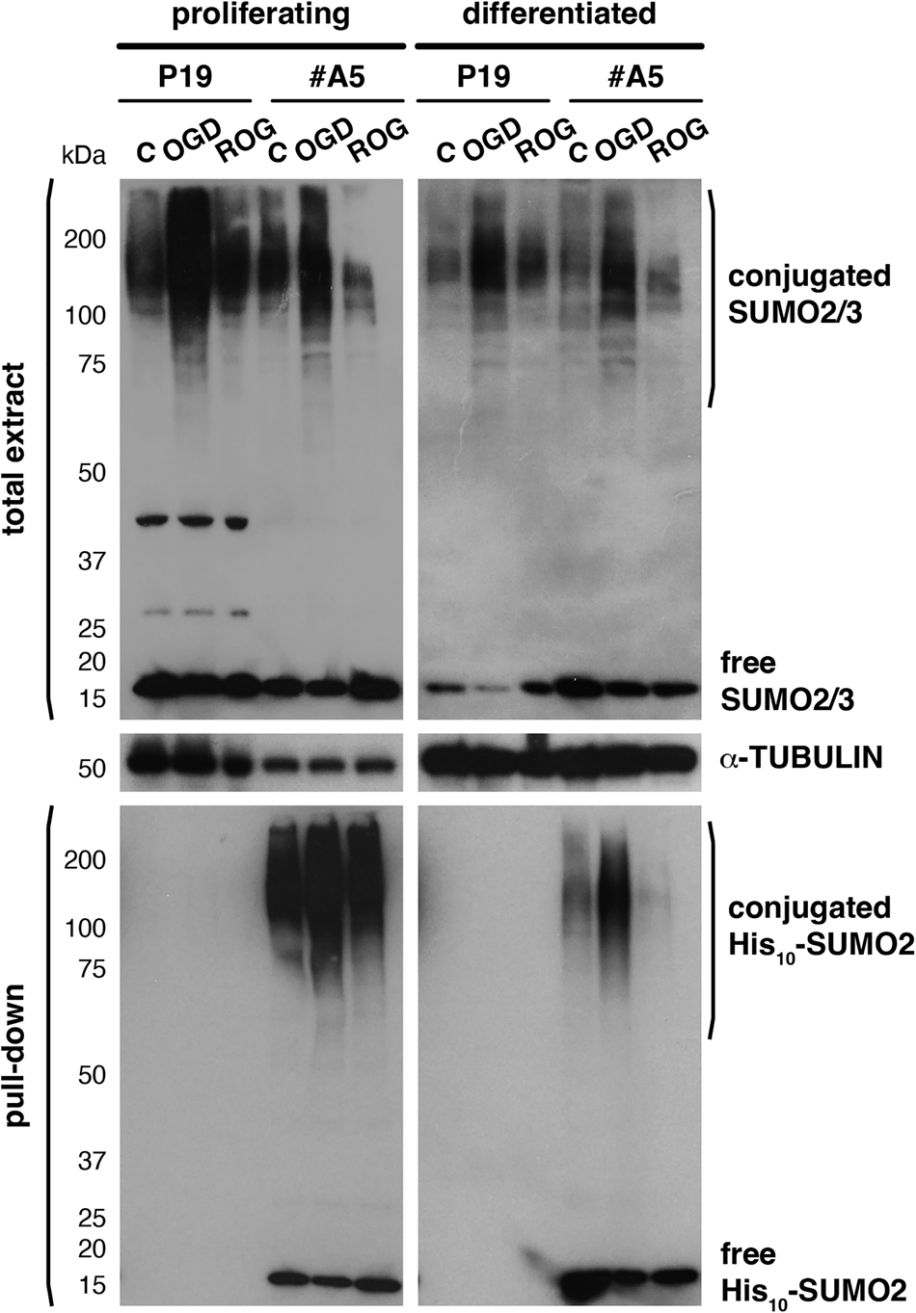
Purification of His_10_-SUMO2 modified proteins under OGD and after ROG in proliferating and differentiated cells. P19 or #A5 cells, proliferating or differentiated into neurons for 7 days, subjected or not (control, C) to 2.5 h of OGD, or to 2.5 h of ROG after 2.5 h of OGD, were used for lysate preparation under denaturing conditions, followed by protein purification with His-bind matrix to precipitate proteins modified by His_10_-SUMO2. Total extracts, as well as pulled-down proteins, were probed by western blot with anti-SUMO2/3 antibodies, which in total extracts detect proteins modified by SUMO2/3 in P19 cells and proteins modified by a mix of SUMO2/3 and His_10_-SUMO2 in #A5 cells, while in pull-down fractions essentially detect His_10_-SUMO2-modified proteins in #A5 cells. Pulled-down proteins were employed for mass spectrometry analysis, in which samples from P19 cells were used as a negative control. For analysis, 3 and 4 independent replicates were prepared for proliferating and differentiated cells, respectively. Selected representative western blots are shown for each condition. Twenty micrograms of total protein were loaded per lane in the case of total extracts. α-TUBULIN was registered as a loading marker in total extracts.

**Fig. 3:**
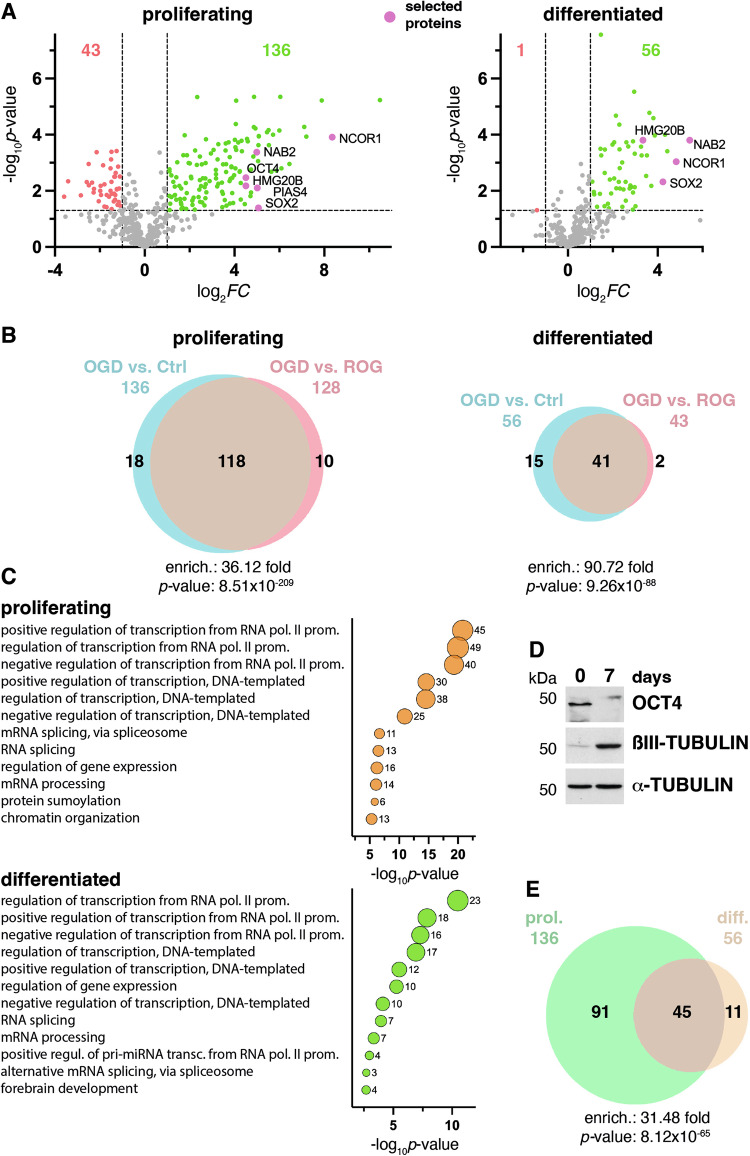
Proteins undergoing increased sumoylation under OGD are related to transcription and RNA processing. **A** Volcano plots of proteins changing their sumoylation state in response to OGD as determined by mass spectrometry analysis in proliferating and differentiated cells. Those proteins out of the established *p*-value and fold change (FC) cutoffs are represented in gray. Proteins undergoing desumoylation are represented in red, while those increasing their sumoylation are represented in green. Selected proteins are highlighted in violet. Numbers represent the number of proteins in each category. **B** Overlapping between proteins undergoing increasing sumoylation in response to OGD (OGD vs. control, Ctrl) and those undergoing desumoylation after ROG (OGD vs. ROG) in proliferating and differentiated cells has been represented by Venn diagrams. **C** Gene ontology (GO) analysis of proteins increasing sumoylation in response to OGD in proliferating and differentiated cells has been represented by bubble graphics. Bubble size represents the number of proteins in each category, also indicated next to each bubble. *p*-value cutoffs of 5 × 10^−6^ and 2 × 10^−3^ were established for proliferating and differentiated cells, respectively. **D** Western blot for checking of proliferating (0 days) and differentiated (7 days) cells by analysis of the pluripotency and differentiation markers OCT4 and ßIII-TUBULIN, respectively; α-TUBULIN was registered as a loading marker. **E** Overlapping between proteins undergoing increasing sumoylation in response to OGD in proliferating (prol.) and differentiated (diff.) cells, represented by Venn diagrams. **B**, **E** Numbers on the top indicate the total number of proteins in each condition. Enrichment (enrich.) of the overlapping, together with the associated *p*-value, as determined by the hypergeometric test, is indicated below.

Extracts from #A5 and parental P19 cells as a control, normally growing (proliferating) or differentiated, were prepared under denaturing conditions. His_10_-SUMO2-modified proteins were purified by pull-down using Ni-NTA beads (Fig. 2), and samples were prepared for mass spectrometry-based proteomics analysis (MS). Heatmaps confirmed the reproducibility of the results in the different replicates after protein purification, and also showed enrichment of a subset of proteins under OGD in comparison with control and ROG conditions in #A5 cells and not in the parental P19 cell line (Supplementary Fig. S2). A total of 670 proteins were consistently identified by MS in at least one condition in proliferating cells, and 434 could be considered sumoylation substrates (Supplementary Dataset S1). We first focused on changes in sumoylation between OGD and control conditions, considering only changes with a *p*-value < 0.05 and a log_2_ fold change (FC) of at least 1. Analysis indicated that 136 proteins increased and 43 decreased their sumoylation level by OGD in proliferating cells (Fig. 3A left). We also compared OGD with ROG and found that the great majority of proteins that increased their sumoylation under OGD (87%), then decreased it after ROG (Fig. 3B left). Gene ontology (GO) analysis of proteins increasing their sumoylation by OGD in proliferating cells indicated that proteins grouped into functional categories mostly related to transcription and RNA processing, but also to sumoylation and chromatin organization (Fig. 3C upper part). If we considered only those proteins that after increasing their sumoylation under OGD decreased it by ROG (118 proteins), GO categories were similar (Supplementary Fig. S3A). On the other hand, from the 43 proteins decreasing their sumoylation by OGD, only 23 (53%) recovered the initial sumoylation state with ROG (Supplementary Fig. S3B). Interestingly, the 43 proteins decreasing their sumoylation by OGD grouped into functional GO categories related to cell cycle regulation, cell differentiation/stemness, and DNA repair (Supplementary Fig. S3C).

### Similar proteins are modified in proliferating and differentiated cells in response to OGD

Taking advantage of the differentiation ability of P19 cells, we aimed to compare proteins changing their sumoylation state by OGD-ROG in proliferating and differentiated cells. The most effective method for P19 differentiation is to treat them with retinoic acid (RA), which gives rise to cells with a neuroectodermal-like phenotype, expressing several neuronal markers [20]. We compared proliferating cells with cells differentiated for 7 days. We checked for efficient differentiation by downregulation of the pluripotency marker OCT4 and induction of the neuronal-specific marker ßIII-TUBULIN (Fig. 3D). After protein purification (Fig. 2), MS and data analysis, we determined that only 56 proteins were increasing their sumoylation level after OGD (Fig. 3A right and Supplementary Dataset S2). As for proliferating cells, most of them (73%), decreased their sumoylation by ROG (Fig. 3B right), and again, similarly to proliferating cells, proteins grouped to GO categories mostly related to transcription and RNA processing but also to forebrain development, supporting the efficiency of the differentiation protocol (Fig. 3C lower part). However, when comparing the proteins with increased sumoylation under OGD in proliferating and differentiated cells, we observed that most of them were the same proteins, i.e., 45 of the 56 proteins (80%) with increased sumoylation under OGD in differentiated cells, also were among the proteins presenting increased sumoylation by OGD in proliferating cells (Fig. 3E). This was also observed when comparing OGD with ROG, 35 of 43 proteins (81%) decreasing their sumoylation by ROG in differentiated cells were in common with proliferating cells (Supplementary Fig. S3D). Thus, the proteins showing altered sumoylation by OGD-ROG in differentiated cells seem to be similar to those identified in proliferating cells.

### SUMO2-modification in response to OGD is confirmed for selected transcription factors and cofactors

Since most of the identified SUMO2 targets are related to transcription we selected transcription-associated proteins for validation, based on medium-high log_2_ FC values and previous identification of target K residues [21–27] (Table 1 and Supplementary Fig. S4A). We chose 6 different proteins for analysis, which included transcription factors and transcription coregulators. On one hand, we selected the transcription factors OCT4 and SOX2, involved in the pluripotency of stem cells but also in differentiation, the sumoylation of which has been already studied [25–27]. On the other hand, we selected a variety of coregulators, the sumoylation of which has been also previously studied. They included: NAB2, a coregulator of the transcription factor KROX20, which depends on NAB2 sumoylation for its development-associated roles, as we have previously described [22]; the high mobility group protein HMG20B, the sumoylation of which we have previously implicated in neuronal differentiation through the Co-REST complex [21]; NCOR1, the ligand-independent corepressor of thyroid-hormone and RA receptors [24]; and PIAS4, a SUMO-specific E3 ligase but also a coregulator of the STAT family and other transcription factors [23, 28]. Because OCT4, as expected, and PIAS4 were not detected in differentiated cells, we validated these proteins, together with SOX2 and NAB2, in proliferating cells, and HMG20B and NCOR1 in differentiated cells.

**Table 1:**
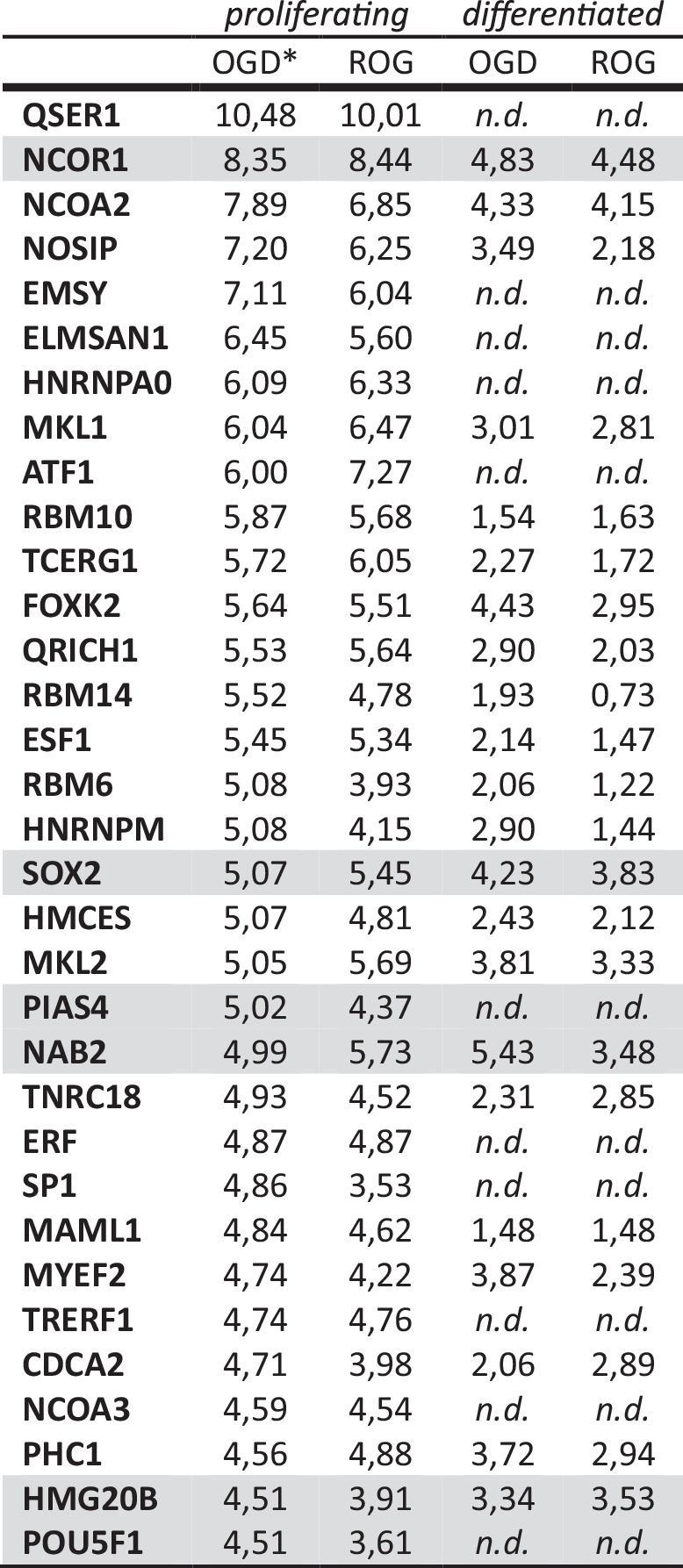
Top-ranking sumoylated proteins.

*n.d.* not detected.

^a^Log_2_ FC values for top-ranking proteins sumoylated under OGD (OGD vs control), and desumoylated after ROG (OGD vs ROG), in proliferating cells. Values for differentiated cells are also included. Validated proteins are shaded in gray. POU5F1, OCT4.

**Fig. 4:**
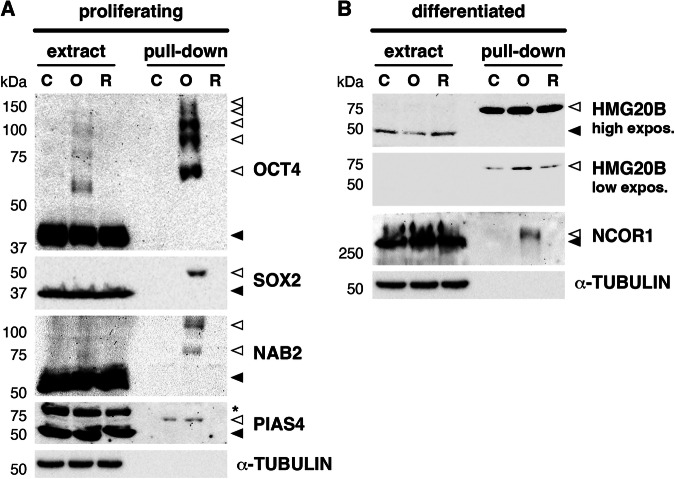
Validation of the sumoylation of selected targets by SUMO2 under OGD and ROG. Validation of proteomic results by selection of several proteins undergoing increased sumoylation under OGD and decreased sumoylation after ROG, as indicated by mass spectrometry, to test them by western blot with specific antibodies against the different proteins, before (extract) and after precipitation of His_10_-SUMO2 with His-bind Matrix (pull-down), in proliferating (**A**) and differentiated (**B**) cells. Twenty micrograms of total protein were loaded per lane in the case of extracts. α-TUBULIN (present in extracts but not after pull-down) was registered as a loading marker. Black arrowheads indicate unmodified proteins while white arrowheads indicate sumoylated products. C control conditions, O OGD, R ROG. *Unspecific band.

As observed in Fig. 4A, sumoylated OCT4 was efficiently pulled down after OGD but not in control or ROG conditions. Sumoylated OCT4 was also faintly visible in extracts, and in both cases appeared as multiple bands, indicative of several SUMO2 molecules modifying OCT4 (Fig.4A). Sumoylated SOX2 also appeared exclusively associated with OGD, but in contrast to OCT4 was observed as a single band (Fig.4A). NAB2 also appeared as several bands, but we have previously demonstrated that this is due to the presence of 2 independent sumoylation sites [22]. Again, sumoylated NAB2 was associated with OGD (Fig. 4A). Conversely, although proteomic data indicated increased sumoylation of PIAS4 associated with OGD, we also detected some sumoylation under control conditions after pull-down, which was removed by ROG (Fig. 4A). In the case of HMG20B, we observed sumoylation under all conditions (Fig. 4B). However, OGD coursed with increased HMG20B sumoylation compared with control or ROG conditions, which also correlated with decreased levels of the unmodified HMG20B protein under OGD (Fig. 4B). Sumoylated NCOR1 was again exclusively detected under OGD (Fig. 4B). Given the high molecular weight of NCOR1, which has been reported to contain several sumoylation sites [24], we cannot distinguish if sumoylated NCOR1 corresponds to multiple bands, coming either from multiple sumoylation sites as from poly-SUMO2 chains, although this is probably the case.

### OCT4 is a SENP7 target for desumoylation after restoring oxygen and glucose

**Fig. 5:**
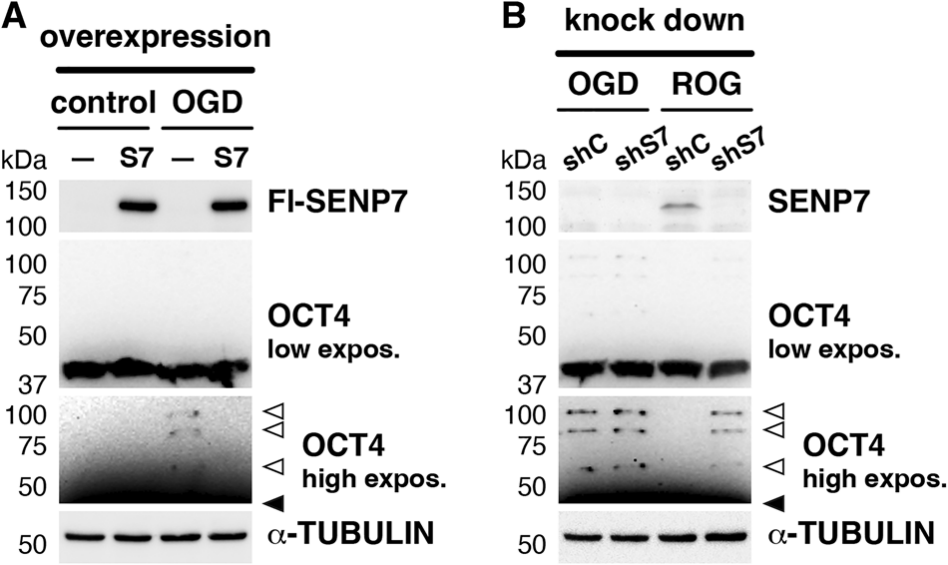
OCT4 is desumoylated by SENP7 when restoring oxygen and glucose. **A** Effect of SENP7 overexpression on OCT4 sumoylation in response to OGD was tested by transfection of a Flag-tagged SENP7 expression construct (S7) or the empty vector (–), under normal growth (control) or OGD conditions. Overexpressed SENP7 was revealed with anti-Flag antibodies. **B** Effect of SENP7 knockdown on OCT4 sumoylation in response to ROG after OGD was tested by transfection of a shRNA construct against SENP7 (shS7) or a control shRNA (shC), under OGD, or after ROG following OGD. Endogenous SENP7 was revealed with anti-SENP7 antibodies. **A**, **B** Twenty micrograms of total protein were loaded per lane. α-TUBULIN was analyzed as a loading marker. High and low exposures (expos.) of the western blot signal for OCT4 are shown for better appreciation of unmodified and modified forms.

It has been reported that mutation of a single K residue in OCT4 efficiently abolishes OCT4 sumoylation [26, 27], and as already indicated, OCT4, in particular, is detected in gels as several migration products when it is sumoylated under OGD (Fig. 4A). These observations, in agreement with the ability of SUMO2 to form poly-SUMO chains, strongly suggest that the several migration products observed correspond to different lengths of a single poly-SUMO2 chain. Because SENP7 has been specifically implicated in editing poly-SUMO2/3 chains [29], and we have previously described that SENP7 is induced by ROG after being depleted under OGD [30], we wondered whether sumoylated OCT4 can be a SENP7 target for desumoylation. To address this question, we decided to conduct gain- and loss-of-function experiments. We first overexpressed a Flag-tagged version of SENP7 in cells subjected to OGD, which resulted in the disappearance of sumoylated forms that were detected under OGD with the empty vector (Fig. 5A). In cells not subjected to OGD (control conditions), overexpressing SENP7 had no consequences on OCT4 sumoylation, as the endogenous protein is expressed and renders OCT4 desumoylated (Fig. 5A). Next, we knocked down the endogenous SENP7 by using a specific shRNA molecule. As observed in Fig. 5B, this molecule did not affect sumoylated OCT4 under OGD, because under these conditions SENP7 is not expressed. However, under ROG, when SENP7 is restored, efficient OCT4 desumoylation is observed when expressing a control shRNA molecule, but not when SENP7 is knocked down by the specific shRNA (Fig. 5B). Thus, sumoylated OCT4 is revealed as a target of SENP7 under ROG conditions.

### Altered sumoylation of selected factors has an impact on cell viability following harmful OGD

To get insight into how the sumoylation of the identified factors impacts the response of cells to OGD, we decided to evaluate cell viability after harmful (20 h) OGD, under overexpression conditions of wild-type (WT) or sumoylation mutant versions of selected proteins. To this end, we chose OCT4, SOX2, NAB2, and PIAS4, and generated sumoylation mutant versions by mutating the previously identified target K residues to R (KR) (Supplementary Fig. S4A). We also checked for similar expression levels of WT and KR versions after transfection of the corresponding constructs (Supplementary Fig. S4B).

**Fig. 6:**
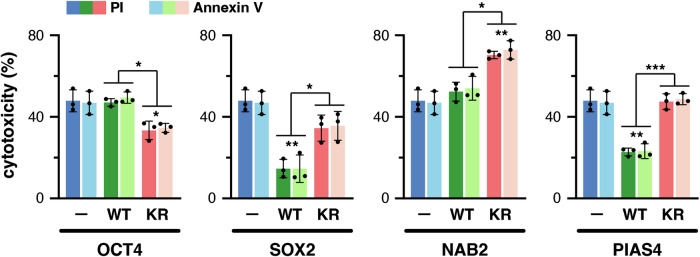
Altered sumoylation of selected factors differentially impacts cell survival after OGD. The percentage of cytotoxicity was estimated in cells subjected to harmful OGD by propidium iodide (PI) incorporation and Annexin V labeling, after transfection of the indicated expression constructs of the indicated proteins: empty vector (control, −), wild type version (WT), or sumoylation mutant (KR). Values are means ± s.d. of three independent determinations. The statistical significance of the differences in cytotoxicity was analyzed by one-way ANOVA (*p* < 0.05) followed by Tukey’s post-test. Differences with the corresponding control are indicated on top of the bars. Other differences between groups of samples are indicated with lines: *p < 0.05, **p < 0.01, ***p < 0.001.

OGD-mediated cytotoxicity was determined by Annexin V/propidium iodide (PI) labeling/incorporation, which reached values of 47% and 48%, respectively, in control conditions using the empty vector, in comparison with around 12% detected after transfection of the empty vector in the absence of OGD (Supplementary Fig. S4C). After subjecting the cells to harmful OGD, we observed that overexpressing an OCT4 mutant unable to be sumoylated resulted in lower toxicity compared with control conditions (empty vector). At the same time, WT overexpression had no effect (Fig. 6). Conversely, overexpression of WT SOX2 resulted in a strong decrease of cell toxicity, while mutant SOX2 slightly reduced toxicity, although not statistically significant (Fig. 6). Regarding NAB2, the WT version had no effect. In contrast, overexpression of the sumoylation mutant resulted in increased cytotoxicity (Fig. 6). Finally, PIAS4 behaved quite similarly to SOX2 since overexpression of the WT version reduced toxicity, while the sumoylation mutant had no impact on cell viability (Fig. 6). Therefore, altered sumoylation of selected factors differentially impacts cell viability when overexpressed. Notably, for all the proteins analyzed, the effects caused by the WT versions were significantly different from those caused by the sumoylation mutants.

## Discussion

Oxygen and glucose are essential for all cell types of the different tissues that make up the human body. Tissue architecture and physiology may cause some cells to be naturally subjected to limited conditions of these nutrients. However, severe deprivation of them has drastic consequences on cell viability. Damage depends not only on the level of deprivation but also on the duration of the insult, and cells need to accurately measure it to make the decision of surviving or inducing cell death to avoid compromising the correct functioning of tissues or organs. Cancer cells inside solid tumors have adapted to OGD conditions, which gives them selective advantages. In all these scenarios, robust and coordinated sensing and signaling mechanisms are required to provide the appropriate response to successfully cope with stress conditions. Almost two decades ago it was observed for the first time a correlation between a drastic reduction in blood flow and increased protein sumoylation in squirrels’ brains during hibernation torpor [13], suggesting that protein sumoylation displays a protective role. Since then, several works have experimentally corroborated this hypothesis in cultured cells and animal models, showing that enhanced protein sumoylation protects cells against ischemia (reviewed in [9]). Most of these works have shown that massive and indiscriminate enhancement of sumoylation results in substantial cell protection. However, a non-selective increase in sumoylation probably limits better outcomes and reduces the effectiveness of a putative therapeutic application. This highlights the need and relevance of identifying specific SUMO targets playing particular roles in cell survival in response to OGD conditions, to selectively act on them to improve cell survival outcomes. Indeed, it is not only important to identify SUMO targets in response to OGD but also to precisely investigate the effects of their increased sumoylation since we cannot exclude the possibility that increased sumoylation of certain targets is detrimental to cell protection.

In this work, we have identified a total of 191 different proteins changing their sumoylation state in response to OGD. The majority of them (77%) experienced increased sumoylation, and of these, 85% were subsequently desumoylated in a few hours when restoring normal growth conditions, supporting that sumoylation is a highly dynamic process. Genes coding for these proteins mainly group to GO categories related to transcription and RNA splicing, indicating that proteins involved in gene expression are immediate SUMO targets in response to OGD. Although previous proteomic studies related to OGD or hypoxia conditions have identified a more limited number of proteins undergoing changes in sumoylation, and the experimental approaches employed differ in some aspects from those used in our work, they have also identified SUMO targets involved in the control of gene expression [15, 16, 31]. Since SUMO has been notably associated with transcription repression [32], we can speculate that increased sumoylation of transcription-related proteins in response to OGD is a way to repress or diminish transcription in the cell under these conditions. This agrees with the previously reported predominant gene downregulation after 4 h of OGD in primary neuron cultures [33]. Also, concerning this, among proteins being sumoylated in response to OGD, we have identified the NAB2 corepressor, which has also been identified as a SUMO target in response to OGD or hypoxia in previous proteomic studies [16, 31], and which we have previously reported to depend on sumoylation to display repressor activity during hindbrain development [22]. Additionally, we identified PIAS4 as a SUMO2 target under OGD, according to a previous report of various PIAS proteins increasing their sumoylation in response to OGD [16]. Of note, it would be interesting to identify SUMO targets in the absence of glucose alone and compare them with those from OGD studies and those previously identified under hypoxia [31].

Remarkably, despite the lower number of proteins identified in differentiated cells, no great differences were observed between the OGD-associated SUMO2 proteome of proliferating and differentiated cells. This indicates that SUMO2 targets in response to OGD are probably general factors regulating common cellular aspects like metabolism, growth, and division, and not cell type-specific processes. This agrees with the fact that OGD is harmful to all cell types and tissues, and it probably triggers the same signaling pathways in all of them to maintain cell viability and activate alternative ways of obtaining energy. On the other hand, in proliferating cells, we have also observed a minority group of proteins undergoing desumoylation in response to OGD. These proteins are grouped in GO categories related to cell cycle, DNA repair, and differentiation/stemness. It is known that SUMO plays relevant roles in cell cycle progression and the maintenance of pluripotency (reviewed in [1]), being particularly required for mitosis [34]. Thus, the desumoylation of key factors involved in these processes can result in their interruption. Of particular interest are proteins SMARCE1, SMARCC1, PHF10, PBRM1, and BRD7 since they form part of the SWI/SNF complex PBAF [35]. SWI/SNF complexes are well-known chromatin remodeling complexes involved in many processes, including cell growth, division, and differentiation. More precisely, PBAF is notably involved in regulating cell differentiation and probably cell-type identity, being also required for the maintenance of genomic integrity during mitosis (reviewed in [36]). PBAF is especially relevant for nervous system development, both for the maintenance of neural progenitors and for the differentiation of post-mitotic cells, and the sumoylation of PHF10 and PBRM1 subunits, in particular, has been linked to complex functionality [37, 38]. It has been proposed that a major role of SUMO is to facilitate complex assembly due to the simultaneous presence of sumoylation sites and non-covalent SUMO interacting motives (SIMs) in various subunits of a complex, which undergo protein-group sumoylation [39]. This is especially evident for chromatin repressor complexes [32], in which the absence of sumoylation may destabilize subunit interactions and complex architecture. Conversely, sumoylation may also destabilize the interaction of certain proteins. For instance, two of the proteins that we have characterized here, OCT4 and SOX2, which operate as a heterodimer to sustain *Nanog* expression for pluripotency maintenance in stem cells, have been described to disassemble when sumoylated [40]. Since we have observed increased sumoylation of both proteins under OGD, we can anticipate heterodimer disassembly under these conditions.

**Fig. 7:**
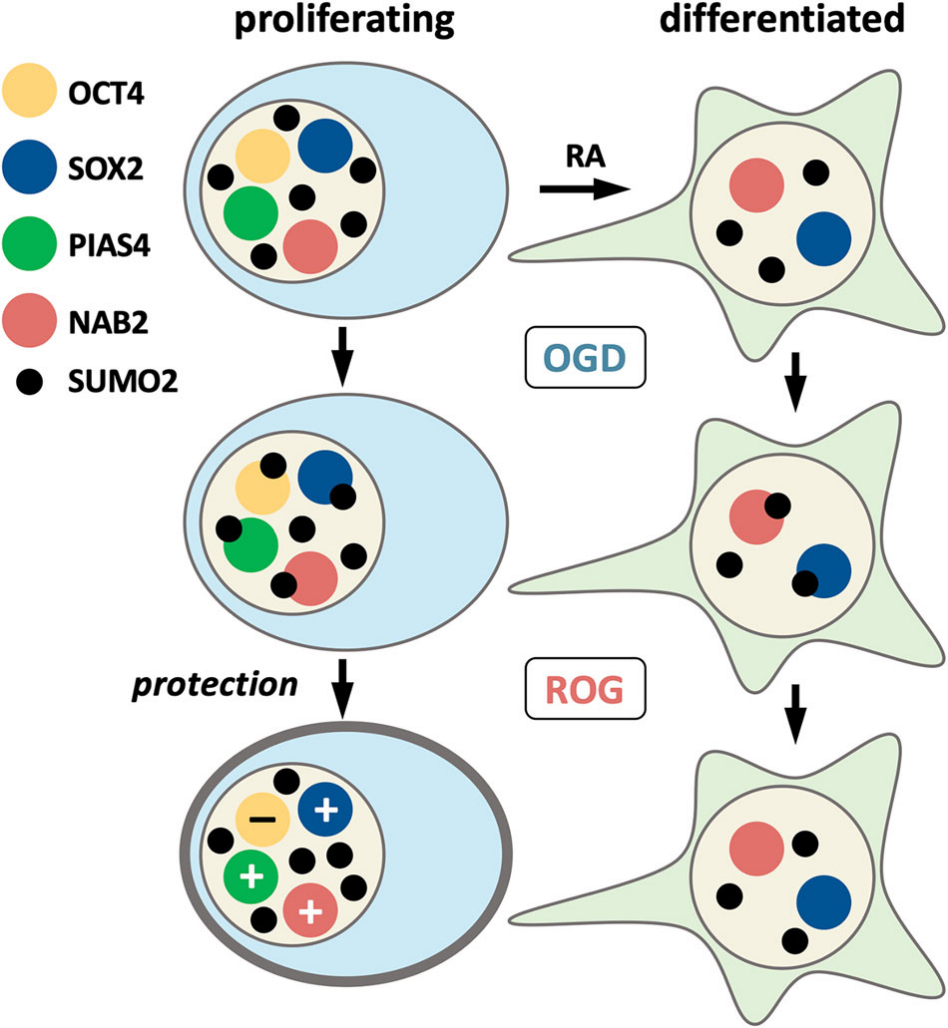
Schematic representation of sumoylation-mediated protection of P19 cells. The cartoon shows increased sumoylation of proteins during OGD and subsequent desumoylation after ROG in proliferating and differentiated P19 cells (RA-treated). Key proteins, the sumoylation of which is important for survival outcomes, are depicted. Less SUMO targets were detected in differentiated cells, but they were similar to those detected in proliferating cells. In the latter, the net balance in terms of cell protection was positive (+) even though modification of some factors is unfavorable to survival (–).

OCT4 and SOX2 have been analyzed, together with NAB2 and PIAS4, for their impact on cell survival when overexpressing WT or sumoylation defective (KR) versions. Our analysis has demonstrated a variety of consequences depending on the protein. In general, overexpression of WT versions either has no effect or is beneficial for cell survival. The beneficial effect of overexpressing PIAS4 is abrogated if its sumoylation is impeded, indicating that PIAS4-mediated survival depends on its sumoylation. SOX2 behaves similarly, although overexpression of its KR mutant has also a positive impact on cell survival, indicating that the beneficial effect of SOX2 is in part independent of sumoylation. Surprisingly, OCT4 behaves differently than SOX2 since it is beneficial only when sumoylation is impeded, suggesting that sumoylation blocks its capacity to promote cell survival and that interfering with it could be favorable for the cells. Sumoylation has been reported to differentially impact the transcription activity of OCT4 and SOX2 [26, 41], which is exemplified in the control of *Nanog* expression [40]. This is not unexpected, since, despite a joint role as a heterodimer in stem cells, these factors also act independently during development [42]. Finally, overexpression of WT NAB2 does not affect cell survival but surprisingly impeding its sumoylation is detrimental to it, indicating that maintaining its sumoylation capacity is necessary for cell protection. Then, these results support the aforementioned need to functionally analyze each SUMO target independently for its role in cell viability. As commented, although the general outcome of indiscriminate increased sumoylation under OGD is positive in terms of cell survival, only a detailed analysis of each sumoylated target will provide information on the best combination of sumoylated and non-sumoylated forms of different proteins to significantly increase protection in harmful conditions and exploit it therapeutically (Fig. 7). Hence, therapies aimed at promoting the sumoylation of PIAS4, NAB2 and SOX2 while promoting the desumoylation of OCT4, through drug-mediated inhibition of specific proteases and ligases, may result in better survival outcomes. It has been shown that the graft in ischemic stroke of neural stem cells displaying enhanced sumoylation due to UBC9 overexpression leads to increased survival and differentiation [43]. Similarly, cells overexpressing WT PIAS4, NAB2, and SOX2, but mutant OCT4, could also show higher survival rates and, therefore, be of therapeutic interest for ischemia.

As SUMO2/3 is the major SUMO form responding to stress conditions and can form poly-SUMO chains [44], we hypothesized and have demonstrated that one of our poly-sumoylated targets under OGD, OCT4, is the substrate for desumoylation after ROG by the SUMO proteases SENP7, specialized in editing poly-SUMO2/3 chains [29]. We have previously implicated SENP7 in promoting cell survival after harmful OGD in tumor cells [30], showing an opposite effect to the SENP3 protease promoting cell death [45, 46]. Besides, SENP1 has also been involved in neuroprotection during transient brain ischemia/reperfusion [47]. Interestingly, both SENP1 and SENP3 have been demonstrated to be catalytically inactivated by hypoxia in a rapid and reversible manner [48], while we have shown reversible downregulation of SENP7 during OGD [30]. All this illustrates the importance of SENP proteases in the sensing and signaling of hypoxia or OGD conditions, which probably participate in measuring the depth of the damage and contribute to making the correct decision between surviving or inducing cell death. Thus, it is probably not the increased sumoylation of proteins per se under OGD that associates with cell protection but the subsequent desumoylation after ROG by specific SENP proteases, which participate in signaling to elaborate the appropriate response to harmful conditions in terms of cell survival.

## Materials and methods

### Cell culture, transfection, and generation of stable cell lines

P19 cells were directly obtained from ATCC (LGC Standards, Barcelona, Spain) as authenticated. They were cultured in Dulbecco’s modified Eagle’s medium (DMEM) (Sigma-Aldrich, St. Louis, MO, USA) supplemented with 10% fetal bovine serum (FBS, Sigma-Aldrich), and 10 ml/l of an antibiotic solution with Penicillin (100 U/ml) and Streptomycin (10 mg/ml) (Sigma-Aldrich). Cells were tested for the absence of mycoplasma. Transfection was performed with Lipofectamine 2000 (Invitrogen, Life Technologies, Paisley, UK), and cells were harvested or subjected to further analysis 24 h later. For knockdown with shRNA-expressing plasmids, cells were subjected to further analysis after 72 h of transfection. For stable cell lines, the integration of plasmid pAV1219 [49], driving the expression of His_10_-SUMO2, was selected in 4 µg/ml puromycin for 10–14 days to the appearance of resistant isolated colonies. Neuronal differentiation was induced by RA at 0.5 µM in normal culture medium containing 5% FBS instead of 10% FBS for 4 days (with a replacement at day 2) in non-adherent plates, followed by 3 additional days in normal medium in adherent plates in the absence of RA. OGD conditions were obtained by incubating the cells at the indicated times in overnight equilibrated normal medium, but based on DMEM lacking glucose (Sigma-Aldrich), in HEPA Class 100 incubators (Thermo Fisher Scientific, Waltham, MA, USA) with 1% oxygen. Harmful OGD for cytotoxicity experiments was obtained by subjecting cells to OGD for 20 h.

### Western blot

For western blot under denaturing conditions, cells were lysed in 8 M urea, 10 mM Tris-HCl pH 8.0 buffer. Lysis buffer for non-denaturing conditions consisted of 50 mM Tris-HCl pH 7.5, 150 mM NaCl, 1% Triton X-100, supplemented with the EDTA-containing complete protease inhibitor cocktail (Sigma-Aldrich). The Bradford reactive assay (Bio-Rad, Hercules, CA, USA) was used for protein quantification. Proteins were separated in SDS-containing polyacrylamide gels and then transferred to PVDF membranes (GE Healthcare, Buckinghamshire, UK) for antibody hybridization. Membranes were subsequently processed with the chemiluminescence ECL system (Bio-Rad) and examined in a ChemiDoc XRS apparatus (Bio-Rad). Antibodies used were as follows: mouse anti-Flag (1:2000, M2, #F1804, Sigma-Aldrich), mouse anti-α-TUBULIN (1:10000, DM1A, #T9026, Sigma-Aldrich), mouse anti-SUMO2/3 (1:2000, 8A2, #ab81371, Abcam, Cambridge, UK), mouse anti-HIF1A (1:5000, #MAB1536, R&D Systems, Minneapolis, MN, USA), mouse anti-His (1:3000, #27471001, GE Healthcare), mouse anti-NAB2 (1:1000, 1C4, #sc-23867, Santa Cruz Btg., Dallas, TX, USA), rabbit anti-OCT4 (1:1000, H-134, #sc-9081, Santa Cruz Btg.), rabbit anti-PIAS4 (1:500, #PA5-20954, Thermo Fisher Scientific), rabbit anti-SOX2 (1:1000, EPR3131, #ab92494, Abcam), mouse anti-HMG20B (1:500, 4.21, #sc-53123, Santa Cruz Btg.), goat anti-NCOR1 (1:500, C-20, #sc-1609, Santa Cruz Btg.), rabbit anti-SENP7 (1:1000, #ab187126, Abcam), goat anti-mouse HRP (1:10000, #A4416, Sigma-Aldrich), goat anti-rabbit HRP (1:10000, #A6154, Sigma-Aldrich), donkey anti-goat HRP (1:10000, #A50-201P, Bethyl Laboratories Inc., Montgomery, TX, USA). Uncropped images of western blots are provided in a Supplementary File.

### Purification of His_10_-SUMO2 conjugates and mass spectrometry sample preparation

His_10_-SUMO2 conjugates were purified as in [50]. Cell pellets from 3 proliferating and 4 differentiated cells replicates were lysed in 10 pellet volumes of guanidine lysis buffer (6 M guanidine-HCl, 100 mM sodium phosphate, and 10 mM Tris, buffered at pH 8.0) and sonicated to promote DNA fragmentation. Next, lysates were supplemented with imidazole to 50 mM and β-mercaptoethanol to 5 mM. Twenty microlitres (dry volume) of pre-washed Ni-NTA agarose beads (QIAGEN, Austin, TX, USA) were added per 1 ml lysate and incubated overnight at 4 °C while rotating. Next, beads were washed for 10 min with 1 ml of the following wash buffers: wash buffer 1 (6 M guanidine-HCl, 0.1% Triton X-100, 10 mM imidazole, 5 mM β-mercaptoethanol, 100 mM sodium phosphate, and 10 mM Tris, pH 8.0), wash buffer 2 (8 M urea, 0.1% Triton X-100, 10 mM imidazole, 5 mM β-mercaptoethanol, 100 mM sodium phosphate, 10 mM Tris, pH 8.0), wash buffer 3 (8 M urea, 10 mM imidazole, 5 mM β-mercaptoethanol, 100 mM sodium phosphate, 10 mM Tris, pH 6.3), wash buffer 4 (8 M urea, 5 mM β-mercaptoethanol, 100 mM sodium phosphate, 10 mM Tris, pH 6.3), two times. After washing, His_10_-SUMO2 conjugates were eluted for 30 min with one bead volume of elution buffer (7 M urea, 500 mM imidazole, 100 mM sodium phosphate, and 10 mM Tris, buffered at pH 7.0). The elution procedure was repeated another two times, and all eluates were pooled and passed through 0.45-μM filters (Ultrafree, Merck (Millipore), Darmstadt, Germany). In the case of the samples corresponding to differentiated cells, samples were heated at 95 °C for 5 min. Next, samples passed through a reduction-alkylation procedure at room temperature consisting of 1 mM dithiothreitol (DTT) for 30 min, 5 mM chloroacetamide for 30 min, and 6 mM DTT for another 30 min. Subsequently, samples were diluted 4 times with 50 mM ammonium bicarbonate, 250 ng Trypsin (V-5111, Promega, Madison WI, USA) added, and incubated overnight still and in the dark. The resulting peptides were purified using C-18 in-house-assembled StageTips [51], Speed Vac, and resuspended in 10 µl of 0.1% formic acid in water.

### Mass spectrometry data acquisition

Mass spectrometry analysis was performed on an EASY-nLC 1000 system (Proxeon, Odense, Denmark) connected to a Q-Exactive Orbitrap (Thermo Fisher Scientific) through a nano-electrospray ion source. The chromatography gradient was performed through reverse phase in a 15 cm in-house packed column with 1.9 μm C18-AQ beads (Reprospher-DE, Pur, Dr. Manish, Ammerbuch-Entringen, Germany). The gradient length was for 95 min from 2% to 30% acetonitrile in 0.1% formic acid, followed by column re-equilibration at a 200 nl/min flow rate. A Top10 data-dependent acquisition mode was applied. The scan range was 400–2000 m/z. Full-scan MS spectra were acquired at a target value of 3 × 10^6^, a resolution of 70,000, and a maximum injection time of 20 ms. The higher-collisional dissociation tandem mass spectra (MS/MS) were recorded at a target value of 1 × 10^5^ with a resolution of 17,500, an isolation window of 2.2 m/z, a normalized collision energy (NCE) of 25, and a maximum injection time of 60 ms. A dynamic exclusion window of 60 s was considered, and ions with charge 1 and >6 were excluded from triggering MS2 analysis.

### Mass spectrometry data analysis

Mass spectrometry RAW data was analyzed using MaxQuant (v. 1.5.5.1) according to [52], with standard settings with the following modifications: Three missed cleavages were allowed for Trypsin/P in proliferating, and four missed for ArgC in differentiated cells from an in-silico predicted *Mus musculus* proteome (Uniprot 10th Oct 2016). In differentiated cells, Carbamyl (K) was added as a variable modification. Label-free quantification (LFQ) was enabled, but not enabled, and the maximum peptide mass was increased to 5000. Match-between-runs was enabled. Statistical analysis of MaxQuant output was performed in the Perseus computational platform [53]. LFQ values were log_2_ transformed, and Contaminants, Reverse, and Only identified by site proteins were removed, as proteins not consistently identified in all replicates in at least one condition as well. Missing values were randomly imputed according to standard settings and 2-tailed *t*-tests performed to compare between conditions. Tables were exported in MS Excel for comprehensive browsing of the data (Supplementary Datasets S1 and S2). The mass spectrometry proteomics data have been deposited to the ProteomeXchange Consortium via the PRIDE [54] partner repository with the dataset identifier PXD056069.

### Plasmids and shRNA molecules

Plasmid pAV1219 was constructed by cloning a His_10_-SUMO2 cDNA fragment obtained by PCR amplification in the *Pst*I-*Xho*I sites of pLV-IRES-Puro [49]. Expression constructs were based on the pAdRSV-Sp plasmid, and those for SENP7, PIAS4, NAB2, and NAB2-KR have been previously described [22, 30]. For OCT4 and SOX2 expression constructs, the complete cDNAs (ATG to STOP) for *Pou5f1* (*Oct4*) and *Sox2* were obtained by PCR amplification from retrotranscribed (High-Capacity cDNA Reverse Transcription Kit, Applied Biosystems, Carlsbad, CA, USA) total RNA isolated from P19 cells (RNeasy kit, QIAGEN), and cloned between the *Nhe*I and *Nsi*I sites of pAdRSV-Sp with a Flag epitope tag at the N-terminus. Defective sumoylation mutants of PIAS4, OCT4, and SOX2 were generated by standard PCR techniques to substitute target K residues (Supplementary Fig. S4A) by R residues. shRNA constructs were based on the pSuper plasmid (OligoEngine, Seattle, WA, USA), and both control shRNA and shRNA against *Senp7* have been previously described [55].

### Cytotoxicity measurement

Cytotoxicity in cells after harmful OGD was evaluated by determination of Anexin V/PI labeling/incorporation by flow cytometry. To this end, we used the FITC Annexin V Apoptosis Detection Kit with PI (Immunostep, Salamanca, Spain), according to the manufacturer’s instructions. After sample processing, they were analyzed in a BD FACSCalibur flow cytometer apparatus (BD Biosciences, San Jose, CA, USA).

### Statistical and additional analyses

Statistical analyses of cytotoxicity experiments were performed with the software Prism 9.5.1 (GraphPad). Graphics show mean values ± s.d. from 3 independent determinations. We used one-way ANOVA (*p* < 0.05) followed by the Tukey post-test for multiple comparisons for statistical analysis of the different groups (*p < 0.05, **p < 0.01, ***p < 0.001). Normal distribution and similar variances were assumed. The significance of overlapping in Venn diagrams was analyzed through the hypergeometric test at https://systems.crump.ucla.edu/hypergeometric/index.php. Venny 2.1 at http://bioinfogp.cnb.csic.es/tools/venny/index.html was used to build Venn diagrams, which were drawn to scale at https://www.meta-chart.com/venn#/display. GO functional categories were analyzed using DAVID [56] at https://david.ncifcrf.gov/tools.jsp.

## Supplementary information

Supplementary Figures

Uncropped Western Blots

Supplementary Dataset S1 - statistical analysis of MS data - proliferating

Supplementary Dataset S2 - statistical analysis of MS data - differentiated

## Data Availability

Proteomic data analysis is provided in Supplementary Datasets S1 and S2. Mass spectrometry data have been deposited to the ProteomeXchange Consortium via the PRIDE partner repository with the dataset identifier PXD056069 (Username: reviewer_pxd056069@ebi.ac.uk, Password: fme7ufOzRODq). Uncropped images of western blots are contained in the Supplementary File uncropped western blots.

## References

[1] Vertegaal ACO. Signalling mechanisms and cellular functions of SUMO. Nat Rev Mol Cell Biol. 2022;23:715–31. 35750927 10.1038/s41580-022-00500-y

[2] Claessens LA, Vertegaal ACO. SUMO proteases: from cellular functions to disease. Trends Cell Biol. 2024;34:901–12. 38326147 10.1016/j.tcb.2024.01.002

[3] Li X, Rasul A, Sharif F, Hassan M. PIAS family in cancer: from basic mechanisms to clinical applications. Front Oncol. 2024;14:1376633. 38590645 10.3389/fonc.2024.1376633 PMC10999569

[4] Lara-Ureña N, Jafari V, Garcia-Dominguez M. Cancer-associated dysregulation of sumo regulators: proteases and ligases. Int J Mol Sci. 2022;23:8012. 35887358 10.3390/ijms23148012 PMC9316396

[5] Yang Y, He Y, Wang X, Liang Z, He G, Zhang P, et al. Protein SUMOylation modification and its associations with disease. Open Biol. 2017;7:170167. 29021212 10.1098/rsob.170167 PMC5666083

[6] Tempe D, Piechaczyk M, Bossis G. SUMO under stress. Biochem Soc Trans. 2008;36:874–8. 18793154 10.1042/BST0360874

[7] Cimarosti H, Lindberg C, Bomholt SF, Ronn LC, Henley JM. Increased protein SUMOylation following focal cerebral ischemia. Neuropharmacology. 2008;54:280–9. 17991493 10.1016/j.neuropharm.2007.09.010

[8] Yang W, Sheng H, Warner DS, Paschen W. Transient global cerebral ischemia induces a massive increase in protein sumoylation. J Cereb Blood Flow Metab. 2008;28:269–79. 17565359 10.1038/sj.jcbfm.9600523

[9] Karandikar P, Gerstl JVE, Kappel AD, Won SY, Dubinski D, Garcia-Segura ME, et al. SUMOtherapeutics for ischemic stroke. Pharmaceuticals. 2023;16:673. 37242456 10.3390/ph16050673 PMC10221934

[10] Cimarosti H, Ashikaga E, Jaafari N, Dearden L, Rubin P, Wilkinson KA, et al. Enhanced SUMOylation and SENP-1 protein levels following oxygen and glucose deprivation in neurones. J Cereb Blood Flow Metab. 2012;32:17–22. 21989481 10.1038/jcbfm.2011.146 PMC3308141

[11] Datwyler AL, Lattig-Tunnemann G, Yang W, Paschen W, Lee SL, Dirnagl U, et al. SUMO2/3 conjugation is an endogenous neuroprotective mechanism. J Cereb Blood Flow Metab. 2011;31:2152–9. 21863037 10.1038/jcbfm.2011.112 PMC3210338

[12] Lee YJ, Castri P, Bembry J, Maric D, Auh S, Hallenbeck JM. SUMOylation participates in induction of ischemic tolerance. J Neurochem. 2009;109:257–67. 19200349 10.1111/j.1471-4159.2009.05957.x PMC2692380

[13] Lee YJ, Miyake S, Wakita H, McMullen DC, Azuma Y, Auh S, et al. Protein SUMOylation is massively increased in hibernation torpor and is critical for the cytoprotection provided by ischemic preconditioning and hypothermia in SHSY5Y cells. J Cereb Blood Flow Metab. 2007;27:950–62. 16955077 10.1038/sj.jcbfm.9600395 PMC2396349

[14] Lee YJ, Mou Y, Maric D, Klimanis D, Auh S, Hallenbeck JM. Elevated global SUMOylation in Ubc9 transgenic mice protects their brains against focal cerebral ischemic damage. PLoS ONE. 2011;6:e25852. 22016779 10.1371/journal.pone.0025852 PMC3189225

[15] Yang W, Sheng H, Thompson JW, Zhao S, Wang L, Miao P, et al. Small ubiquitin-like modifier 3-modified proteome regulated by brain ischemia in novel small ubiquitin-like modifier transgenic mice: putative protective proteins/pathways. Stroke. 2014;45:1115–22. 24569813 10.1161/STROKEAHA.113.004315 PMC3966925

[16] Yang W, Thompson JW, Wang Z, Wang L, Sheng H, Foster MW, et al. Analysis of oxygen/glucose-deprivation-induced changes in SUMO3 conjugation using SILAC-based quantitative proteomics. J Proteome Res. 2012;11:1108–17. 22082260 10.1021/pr200834f PMC3628696

[17] Kanungo J. Tumor suppressors and endodermal differentiation of P19 embryonic stem cells. Cell Dev Biol. 2015;4:e183. 10.4172/2168-9296.1000e138 PMC4939905 27413642

[18] McBurney MW. P19 embryonal carcinoma cells. Int J Dev Biol. 1993;37:135–40. 8507558

[19] Hendriks IA, D’Souza RC, Yang B, Verlaan-de Vries M, Mann M, Vertegaal AC. Uncovering global SUMOylation signaling networks in a site-specific manner. Nat Struct Mol Biol. 2014;21:927–36. 25218447 10.1038/nsmb.2890 PMC4259010

[20] Correa-Vazquez JF, Juarez-Vicente F, Garcia-Gutierrez P, Barysch SV, Melchior F, Garcia-Dominguez M. The Sumo proteome of proliferating and neuronal-differentiating cells reveals Utf1 among key Sumo targets involved in neurogenesis. Cell Death Dis. 2021;12:305. 33753728 10.1038/s41419-021-03590-2 PMC7985304

[21] Ceballos-Chavez M, Rivero S, Garcia-Gutierrez P, Rodriguez-Paredes M, Garcia-Dominguez M, Bhattacharya S, et al. Control of neuronal differentiation by sumoylation of BRAF35, a subunit of the LSD1-CoREST histone demethylase complex. Proc Natl Acad Sci USA. 2012;109:8085–90. 22570500 10.1073/pnas.1121522109 PMC3361438

[22] Garcia-Gutierrez P, Juarez-Vicente F, Gallardo-Chamizo F, Charnay P, Garcia-Dominguez M. The transcription factor Krox20 is an E3 ligase that sumoylates its Nab coregulators. EMBO Rep. 2011;12:1018–23. 21836637 10.1038/embor.2011.152 PMC3185338

[23] Ihara M, Yamamoto H, Kikuchi A. SUMO-1 modification of PIASy, an E3 ligase, is necessary for PIASy-dependent activation of Tcf-4. Mol Cell Biol. 2005;25:3506–18. 15831457 10.1128/MCB.25.9.3506-3518.2005 PMC1084305

[24] Tiefenbach J, Novac N, Ducasse M, Eck M, Melchior F, Heinzel T. SUMOylation of the corepressor N-CoR modulates its capacity to repress transcription. Mol Biol Cell. 2006;17:1643–51. 16421255 10.1091/mbc.E05-07-0610 PMC1415330

[25] Tsuruzoe S, Ishihara K, Uchimura Y, Watanabe S, Sekita Y, Aoto T, et al. Inhibition of DNA binding of Sox2 by the SUMO conjugation. Biochem Biophys Res Commun. 2006;351:920–6. 17097055 10.1016/j.bbrc.2006.10.130

[26] Wei F, Scholer HR, Atchison ML. Sumoylation of Oct4 enhances its stability, DNA binding, and transactivation. J Biol Chem. 2007;282:21551–60. 17525163 10.1074/jbc.M611041200

[27] Zhang Z, Liao B, Xu M, Jin Y. Post-translational modification of POU domain transcription factor Oct-4 by SUMO-1. FASEB J. 2007;21:3042–51. 17496161 10.1096/fj.06-6914com

[28] Schmidt D, Muller S. PIAS/SUMO: new partners in transcriptional regulation. Cell Mol Life Sci. 2003;60:2561–74. 14685683 10.1007/s00018-003-3129-1 PMC11138616

[29] Shen LN, Geoffroy MC, Jaffray EG, Hay RT. Characterization of SENP7, a SUMO-2/3-specific isopeptidase. Biochem J. 2009;421:223–30. 19392659 10.1042/BJ20090246

[30] Gallardo-Chamizo F, Lara-Urena N, Correa-Vazquez JF, Reyes JC, Gauthier BR, Garcia-Dominguez M. SENP7 overexpression protects cancer cells from oxygen and glucose deprivation and associates with poor prognosis in colon cancer. Genes Dis. 2022;9:1419–22. 36157488 10.1016/j.gendis.2022.02.019 PMC9485274

[31] Chachami G, Stankovic-Valentin N, Karagiota A, Basagianni A, Plessmann U, Urlaub H, et al. Hypoxia-induced changes in SUMO conjugation affect transcriptional regulation under low oxygen. Mol Cell Proteom. 2019;18:1197–209. 10.1074/mcp.RA119.001401 PMC6553927 30926672

[32] Garcia-Dominguez M, Reyes JC. SUMO association with repressor complexes, emerging routes for transcriptional control. Biochim Biophys Acta. 2009;1789:451–9. 19616654 10.1016/j.bbagrm.2009.07.001

[33] Guo S, Tjarnlund-Wolf A, Deng W, Tejima-Mandeville E, Lo LJ, Xing C, et al. Comparative transcriptome of neurons after oxygen-glucose deprivation: Potential differences in neuroprotection versus reperfusion. J Cereb Blood Flow Metab. 2018;38:2236–50. 30152713 10.1177/0271678X18795986 PMC6282217

[34] Mukhopadhyay D, Dasso M. The SUMO pathway in mitosis. Adv Exp Med Biol. 2017;963:171–84. 28197912 10.1007/978-3-319-50044-7_10 PMC11166265

[35] Yuan J, Chen K, Zhang W, Chen Z. Structure of human chromatin-remodelling PBAF complex bound to a nucleosome. Nature. 2022;605:166–71. 35477757 10.1038/s41586-022-04658-5

[36] Hodges C, Kirkland JG, Crabtree GR. The many roles of BAF (mSWI/SNF) and PBAF complexes in cancer. Cold Spring Harb Perspect Med. 2016;6:a026930. 27413115 10.1101/cshperspect.a026930 PMC4968166

[37] Brechalov AV, Georgieva SG, Soshnikova NV. Mammalian cells contain two functionally distinct PBAF complexes incorporating different isoforms of PHF10 signature subunit. Cell Cycle. 2014;13:1970–9. 24763304 10.4161/cc.28922 PMC4111760

[38] Rivera O, Sharma M, Dagar S, Shahani N, Ramlrez-Jarquln UN, Crynen G, et al. Rhes, a striatal enriched protein, regulates post-translational small-ubiquitin-like-modifier (SUMO) modification of nuclear proteins and alters gene expression. Cell Mol Life Sci. 2024;81:169. 38589732 10.1007/s00018-024-05181-8 PMC11001699

[39] Jentsch S, Psakhye I. Control of nuclear activities by substrate-selective and protein-group SUMOylation. Annu Rev Genet. 2013;47:167–86. 24016193 10.1146/annurev-genet-111212-133453

[40] Wu Y, Guo Z, Wu H, Wang X, Yang L, Shi X, et al. SUMOylation represses Nanog expression via modulating transcription factors Oct4 and Sox2. PLoS ONE. 2012;7:e39606. 22745796 10.1371/journal.pone.0039606 PMC3382131

[41] Marelli E, Hughes J, Scotting PJ. SUMO-dependent transcriptional repression by Sox2 inhibits the proliferation of neural stem cells. PLoS ONE. 2024;19:e0298818. 38507426 10.1371/journal.pone.0298818 PMC10954124

[42] Rizzino A, Wuebben EL. Sox2/Oct4: a delicately balanced partnership in pluripotent stem cells and embryogenesis. Biochim Biophys Acta. 2016;1859:780–91. 26992828 10.1016/j.bbagrm.2016.03.006

[43] Bernstock JD, Peruzzotti-Jametti L, Leonardi T, Vicario N, Ye D, Lee YJ, et al. SUMOylation promotes survival and integration of neural stem cell grafts in ischemic stroke. EBioMedicine. 2019;42:214–24. 30905846 10.1016/j.ebiom.2019.03.035 PMC6491415

[44] Enserink JM. Sumo and the cellular stress response. Cell Div. 2015;10:4. 26101541 10.1186/s13008-015-0010-1 PMC4476178

[45] Guo C, Hildick KL, Luo J, Dearden L, Wilkinson KA, Henley JM. SENP3-mediated deSUMOylation of dynamin-related protein 1 promotes cell death following ischaemia. EMBO J. 2013;32:1514–28. 23524851 10.1038/emboj.2013.65 PMC3671254

[46] Zhao S, Xu Z, Niu X, Cao C, Gu Y, Wang H, et al. The role of SUMO specific peptidase 3 in secondary inflammation of ischemic stroke in mice. Biochim Biophys Acta Mol Basis Dis. 2024;1870:167104. 38437993 10.1016/j.bbadis.2024.167104

[47] Zhang H, Wang Y, Zhu A, Huang D, Deng S, Cheng J, et al. SUMO-specific protease 1 protects neurons from apoptotic death during transient brain ischemia/reperfusion. Cell Death Dis. 2016;7:e2484. 27882949 10.1038/cddis.2016.290 PMC5260881

[48] Kunz K, Wagner K, Mendler L, Holper S, Dehne N, Muller S. SUMO signaling by hypoxic inactivation of SUMO-specific isopeptidases. Cell Rep. 2016;16:3075–86. 27626674 10.1016/j.celrep.2016.08.031

[49] Vyas R, Kumar R, Clermont F, Helfricht A, Kalev P, Sotiropoulou P, et al. RNF4 is required for DNA double-strand break repair in vivo. Cell Death Differ. 2013;20:490–502. 23197296 10.1038/cdd.2012.145 PMC3569989

[50] Hendriks IA, Vertegaal AC. A high-yield double-purification proteomics strategy for the identification of SUMO sites. Nat Protoc. 2016;11:1630–49. 27560170 10.1038/nprot.2016.082

[51] Rappsilber J, Mann M, Ishihama Y. Protocol for micro-purification, enrichment, pre-fractionation and storage of peptides for proteomics using StageTips. Nat Protoc. 2007;2:1896–906. 17703201 10.1038/nprot.2007.261

[52] Tyanova S, Temu T, Cox J. The MaxQuant computational platform for mass spectrometry-based shotgun proteomics. Nat Protoc. 2016;11:2301–19. 27809316 10.1038/nprot.2016.136

[53] Tyanova S, Temu T, Sinitcyn P, Carlson A, Hein MY, Geiger T, et al. The Perseus computational platform for comprehensive analysis of (prote)omics data. Nat Methods. 2016;13:731–40. 27348712 10.1038/nmeth.3901

[54] Perez-Riverol Y, Bai J, Bandla C, Garcia-Seisdedos D, Hewapathirana S, Kamatchinathan S, et al. The PRIDE database resources in 2022: a hub for mass spectrometry-based proteomics evidences. Nucleic Acids Res. 2022;50:D543–D52. 34723319 10.1093/nar/gkab1038 PMC8728295

[55] Juarez-Vicente F, Luna-Pelaez N, Garcia-Dominguez M. The Sumo protease Senp7 is required for proper neuronal differentiation. Biochim Biophys Acta. 2016;1863:1490–8. 27039038 10.1016/j.bbamcr.2016.03.028

[56] Huang da W, Sherman BT, Lempicki RA. Systematic and integrative analysis of large gene lists using DAVID bioinformatics resources. Nat Protoc. 2009;4:44–57. 19131956 10.1038/nprot.2008.211

