# Supplementary material for: SUMO2/3 modification of transcription-associated proteins controls cell viability in response to oxygen and glucose deprivation-mediated stress
Source: Cell Death Discov. 2025 May 10;11:230. doi: 10.1038/s41420-025-02513-w (PMC12065886; doi:10.1038/s41420-025-02513-w)
Supplement: Supplementary file 1 — Supplementary Figures [file 41420_2025_2513_MOESM1_ESM.pdf]

Supplementary Figures

SUMO2/3 modification of transcription-associated proteins controls cell viability in response to oxygen and glucose deprivation-mediated stress

Francisco Gallardo-Chamizo[1], Román González-Prieto[1,2], Vahid Jafari[1], Noelia Luna-Peláez[1], Alfred C. O. Vertegaal[2] and Mario García-Domínguez[1]

[1]Andalusian Centre for Molecular Biology and Regenerative Medicine-CABIMER, CSIC-Universidad de Sevilla-Universidad Pablo de Olavide, Av. Américo Vespucio 24, 41092 Seville, Spain
[2]Department of Cell and Chemical Biology, Leiden University Medical Center, Leiden, Netherlands.

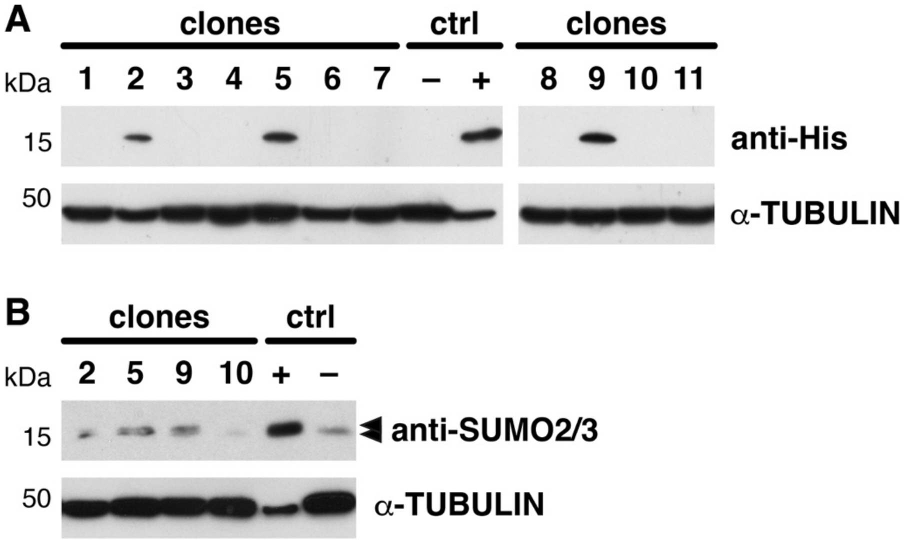

Supplementary Fig. S1 Generation of stable cell lines expressing His_{10}-SUMO2 from P19 cells. A Clones resistant to puromycin after introducing an expression construct for His_{10}-SUMO2 were tested by western blot with anti-His antibodies for stable integration of the construct. **B** Positive clones were subsequently tested with anti-SUMO2/3 antibodies. Arrowheads show a double band for SUMO2/3 corresponding to endogenous SUMO2/3 (lower band) and stably expressed His_{10}-SUMO2 (upper band) in the analyzed clones but not in the controls. **A, B** Positive (+) and negative (-) control (ctrl) samples, corresponding to cells transiently transfected with the expression construct or not transfected, respectively, were also analyzed. 20 µg of total protein were loaded per lane. α-TUBULIN was analyzed as a loading marker.

A

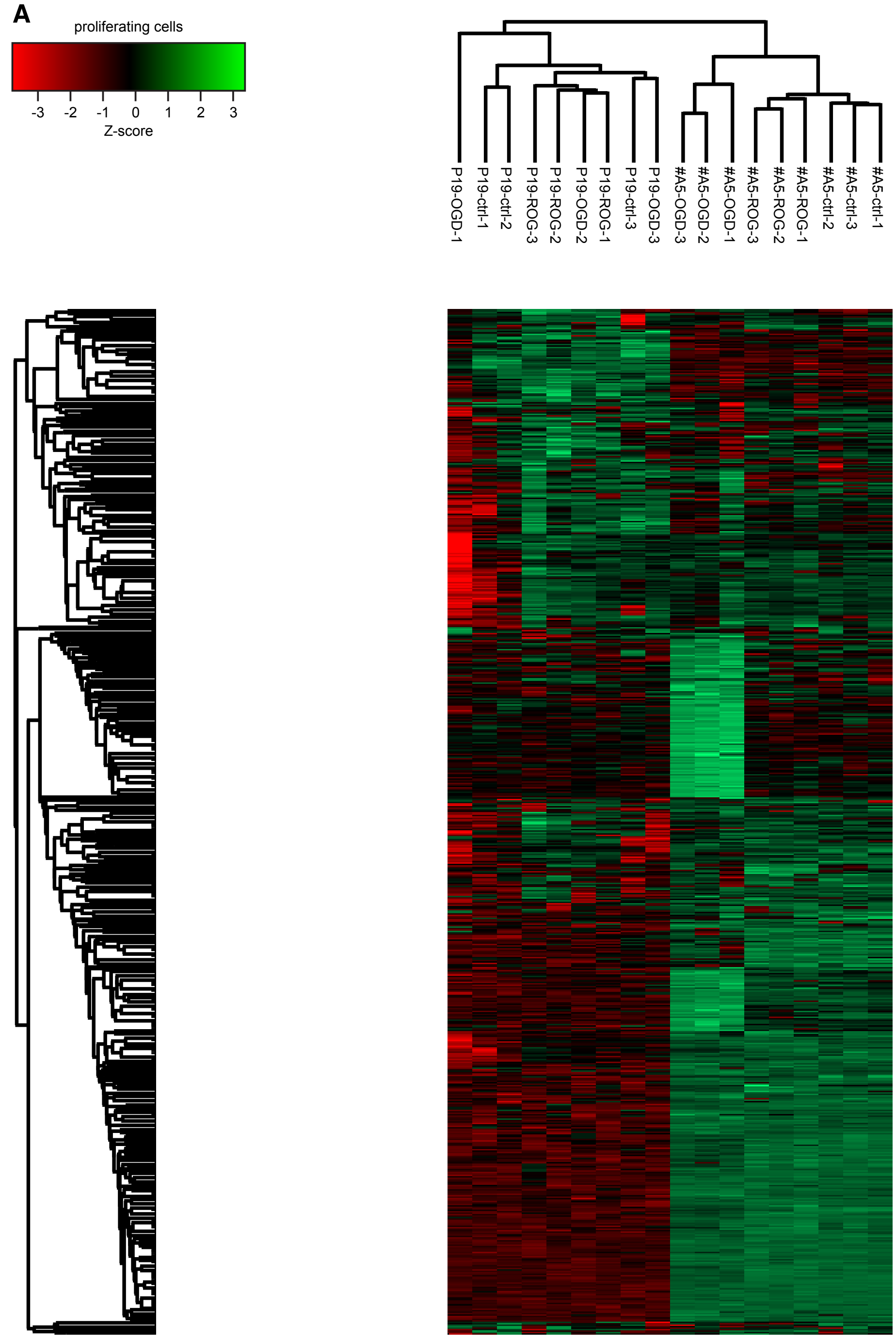

proliferating cells
-3
-2
-1
0
1
2
3
Z-score
P19-OGD-1
P19-ctrl-1
P19-ctrl-2
P19-ROG-3
P19-ROG-2
P19-OGD-2
P19-ROG-1
P19-ctrl-3
P19-OGD-3
#A5-OGD-3
#A5-OGD-2
#A5-OGD-1
#A5-ROG-3
#A5-ROG-2
#A5-ROG-1
#A5-ctrl-2
#A5-ctrl-3
#A5-ctrl-1

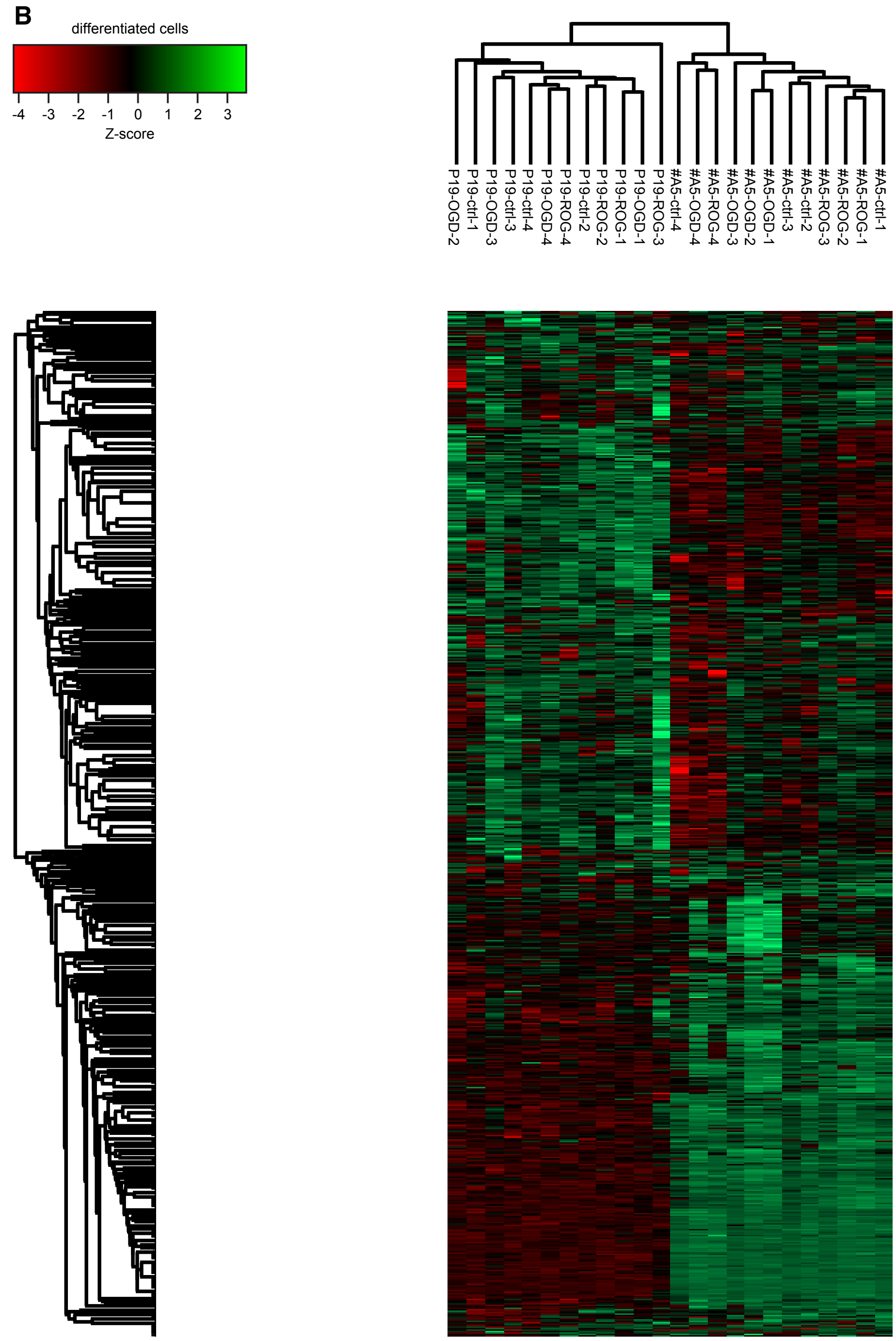

Supplementary Fig. S2 Heatmaps of His$_{10}$-SUMO2 modified proteins under OGD and ROG in proliferating and differentiated cells. Heatmaps of sample clustering after mass spectrometry data analysis of proliferating (**A**) and differentiated (**B**) P19 and #A5 cells are shown. Color code for Z-score values of each heatmap is also indicated.

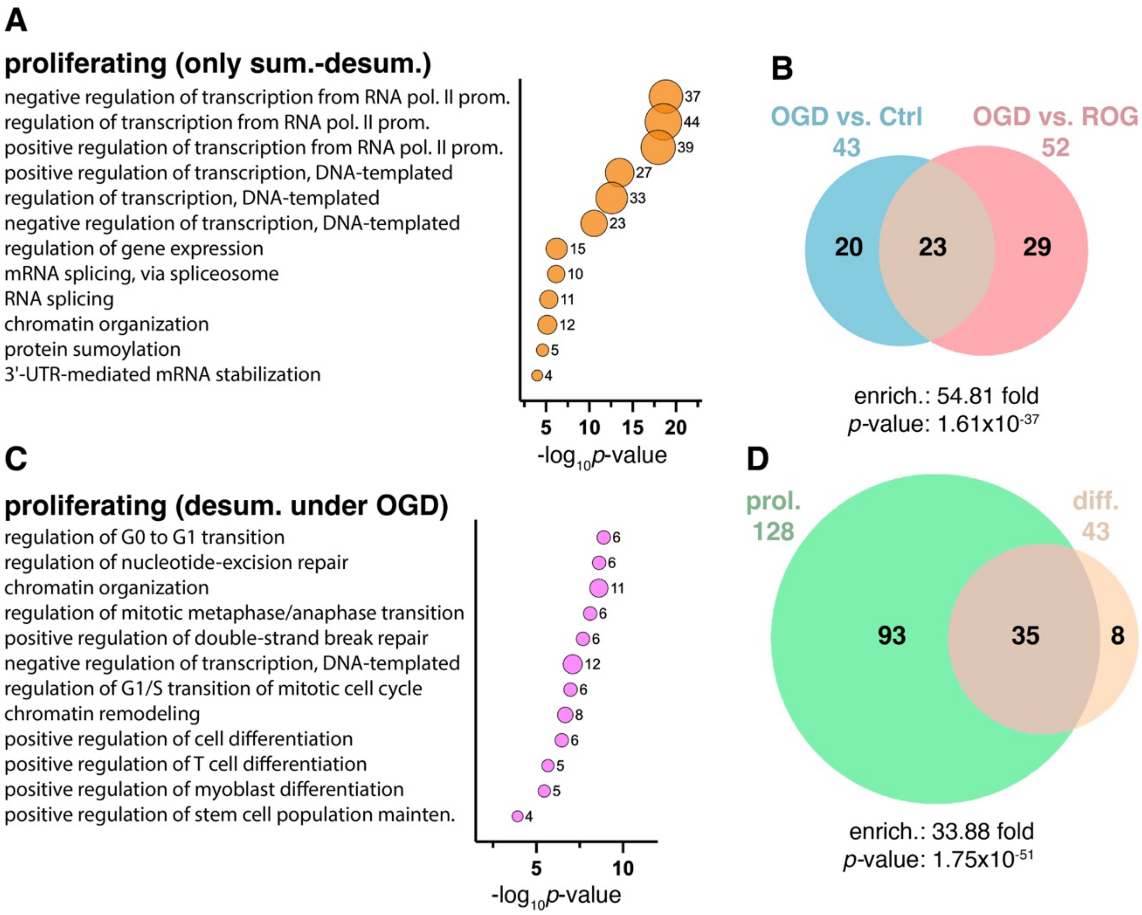

Supplementary Fig. S3 GO analysis of proteins experiencing sumoylation changes in response to OGD and ROG. A Gene ontology (GO) analysis of proteins increasing sumoylation in response to OGD in proliferating cells and subsequently decreasing it after ROG has been represented by bubble graphics. **B** Overlapping between proteins undergoing desumoylation in response to OGD (OGD vs. control, Ctrl) and those undergoing increasing sumoylation after ROG (OGD vs. ROG) in proliferating cells, has been represented by Venn diagrams. **C**) Gene ontology (GO) analysis of proteins decreasing sumoylation in response to OGD in proliferating cells has been represented by bubble graphics. **A**, **C** Bubble size represents the number of proteins in each category, also indicated next to each bubble. *p*-value cutoff $2x10^{-4}$. **D** Overlapping between proteins undergoing decreased sumoylation under ROG after OGD in proliferating (prol.) and differentiated (diff.) cells, has been represented by Venn diagrams. **B**, **D** Numbers on the top indicate the total number of proteins in each condition. Enrichment (enrich.) of the overlapping, together with the associated *p*-value, as determined by the hypergeometric test, is indicated below.

A

Protein	mutation	sequence	references
OCT4	K123R	DGASPEPCTVTPGAVKLEKEKLEQNPEESQD	[26, 27]
SOX2	K247R	QGTPGMALGSMGSVVKSEASSSPPVVTSSSH	[25]
NAB2	K379R	RLHSEELGGPPLKKLKQEVGEQSHNEIQQPP	[22]
	K517R	PGPHPALVEGRRSSVKVEAEASRQ*	
PIAS4	K35R	LQMLLGFVGRSKSGLKHELVTRALQLVQFDC	[23]

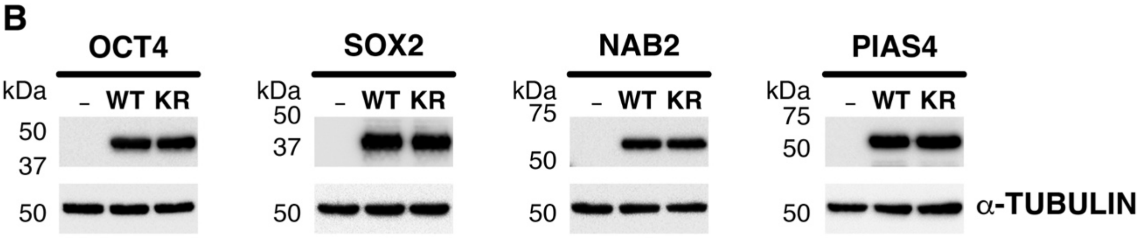

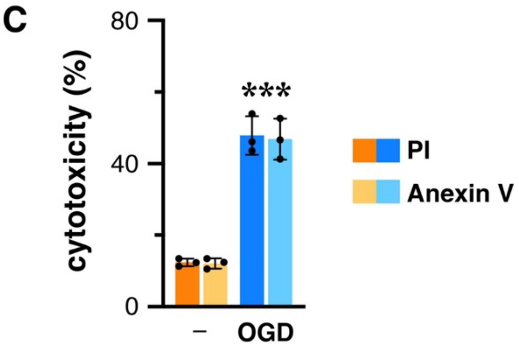

Supplementary Fig. S4 Sumoylation mutants of selected factors for cytotoxicity analysis in response to OGD. A Schematic representation of the mutated Lys residue (K, red) and the surrounding amino acid sequence of the indicated proteins. References previously describing these mutations are also indicated. **B** Comparison of expression levels between wild-type (WT) and sumoylation mutant (KR) versions of the indicated proteins tagged with a Flag epitope at the N-terminus, determined in P19 cells by western blot with anti-Flag antibodies. As a negative control, extracts from cells transfected with empty vector (–) have also been analyzed. 20 µg of total protein were loaded per lane. α-TUBULIN was registered as a loading marker. **C** The percentage of cytotoxicity was determined in proliferating cells transfected with the empty vector used for protein expression constructs (pAdRSV-Sp) subjected or not (control, –) to harmful OGD, by propidium iodide incorporation and Anexin V labeling. Values are means ± s.d. of three independent determinations. The statistical significance of the differences in cytotoxicity was analyzed by one-way ANOVA ($p < 0.05$) followed by Tukey's post-test. Differences with the corresponding controls are indicated on top of the bars. ***$p < 0.001$.
