# Supplementary material for: SUMO2/3 modification of transcription-associated proteins controls cell viability in response to oxygen and glucose deprivation-mediated stress
Source: Cell Death Discov. 2025 May 10;11:230. doi: 10.1038/s41420-025-02513-w (PMC12065886; doi:10.1038/s41420-025-02513-w)
Supplement: Supplementary file 2 — Uncropped Western Blots [file 41420_2025_2513_MOESM2_ESM.pdf]

Supplementary File. *Uncropped western blots*

Uncropped western blots for main and Supplementary Figures. Boxes indicate the area that is shown for each panel of the different figures.

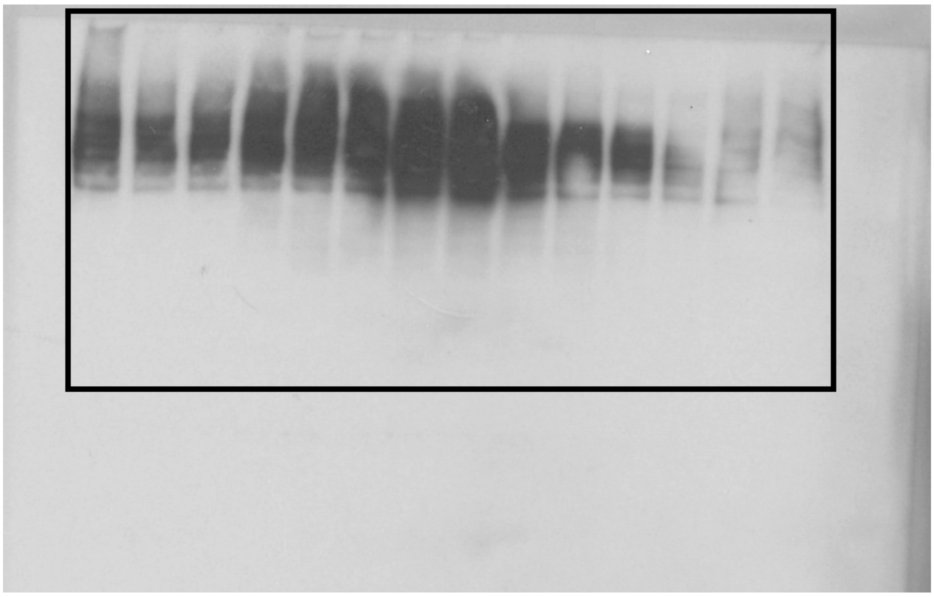

conjugated SUMO2/3

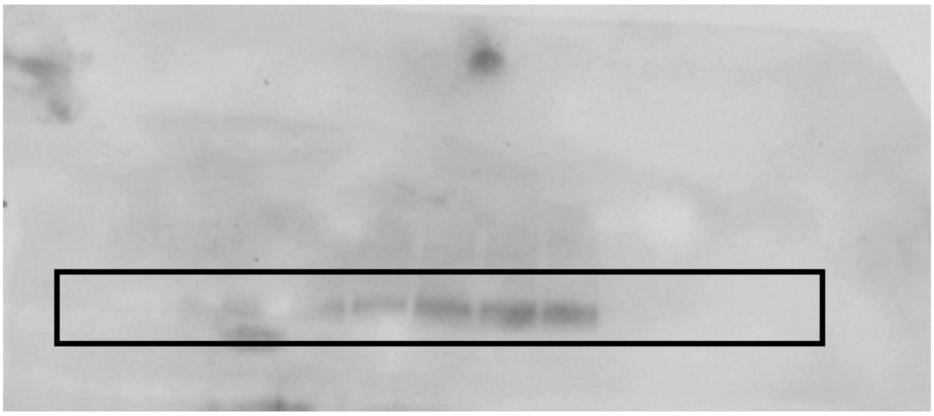

HIF1α

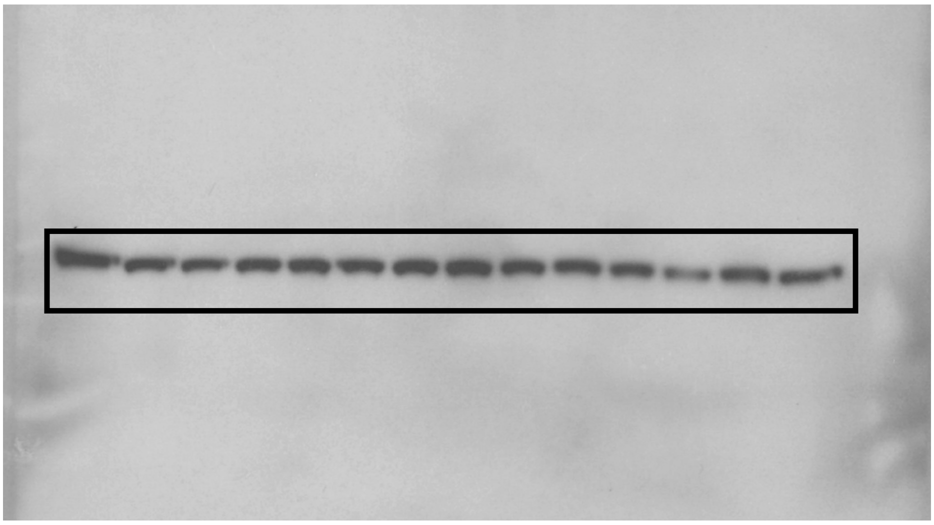

α-TUBULIN

Corresponds to Fig. 1A

Uncropped western blots

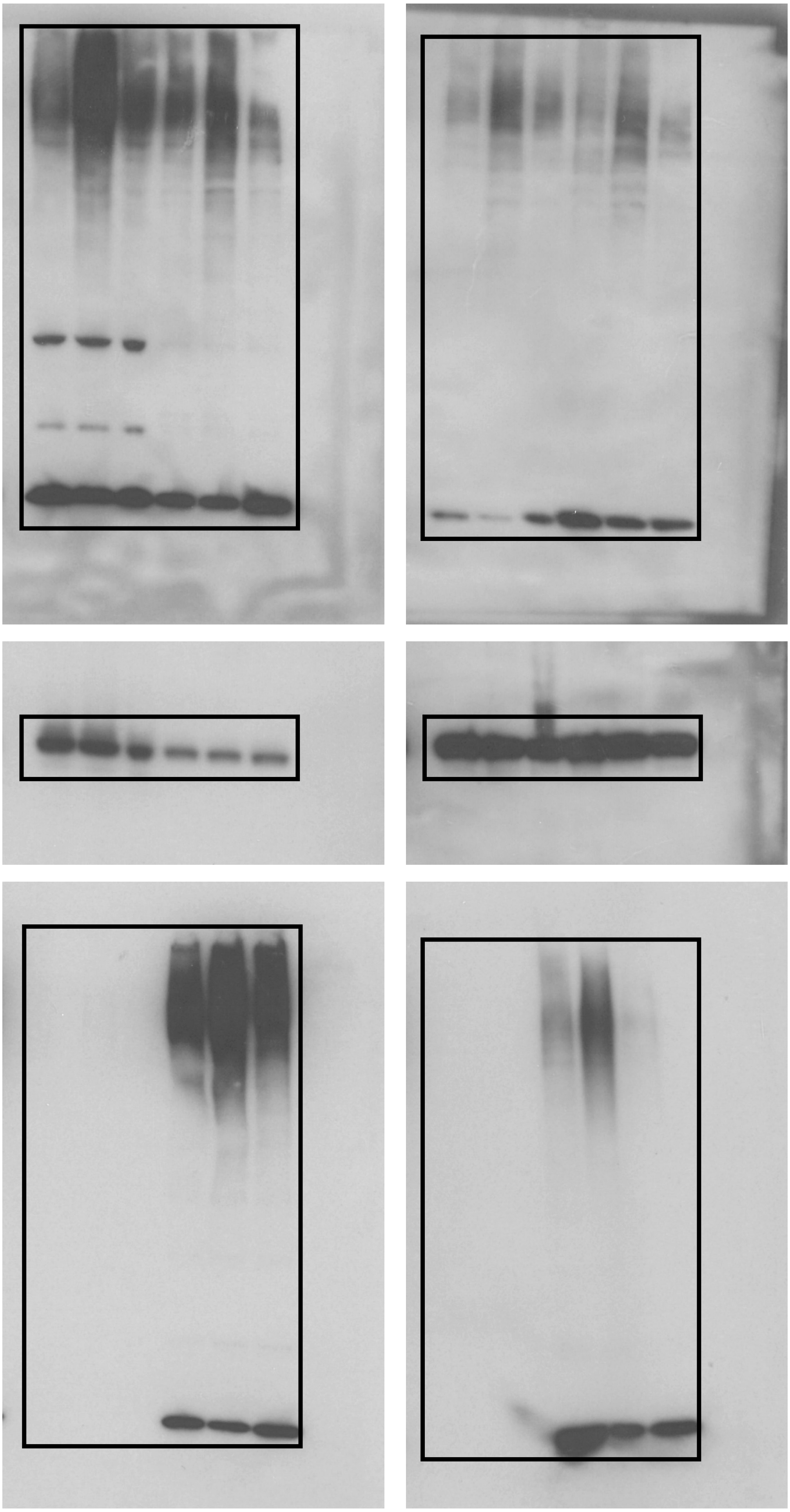

conjugated SUMO2/3

free SUMO2/3

α-TUBULIN

conjugated His_{10}-SUMO2

free His_{10}-SUMO2

Corresponds to Fig. 2

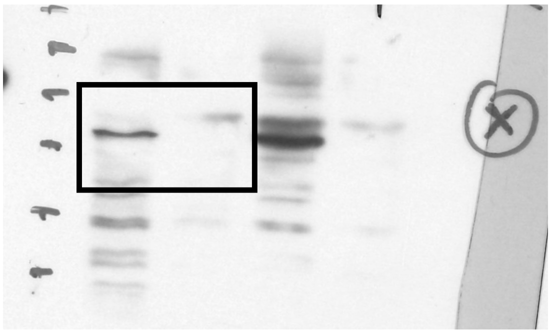

OCT4

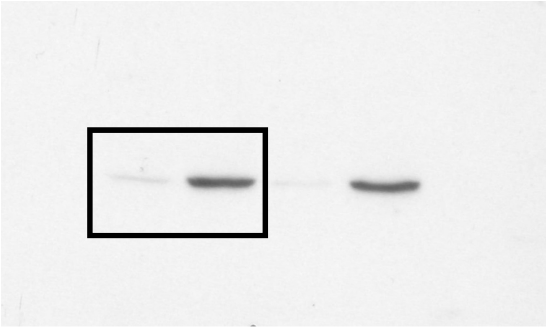

ßIII-TUBULIN

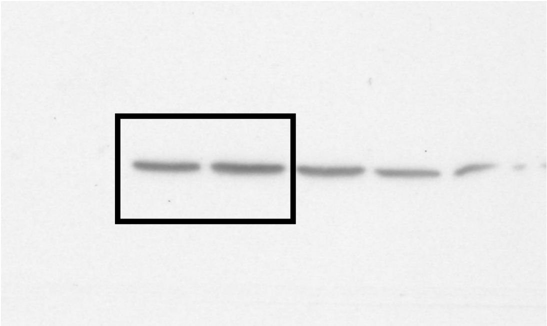

α-TUBULIN

Corresponds to Fig. 3D

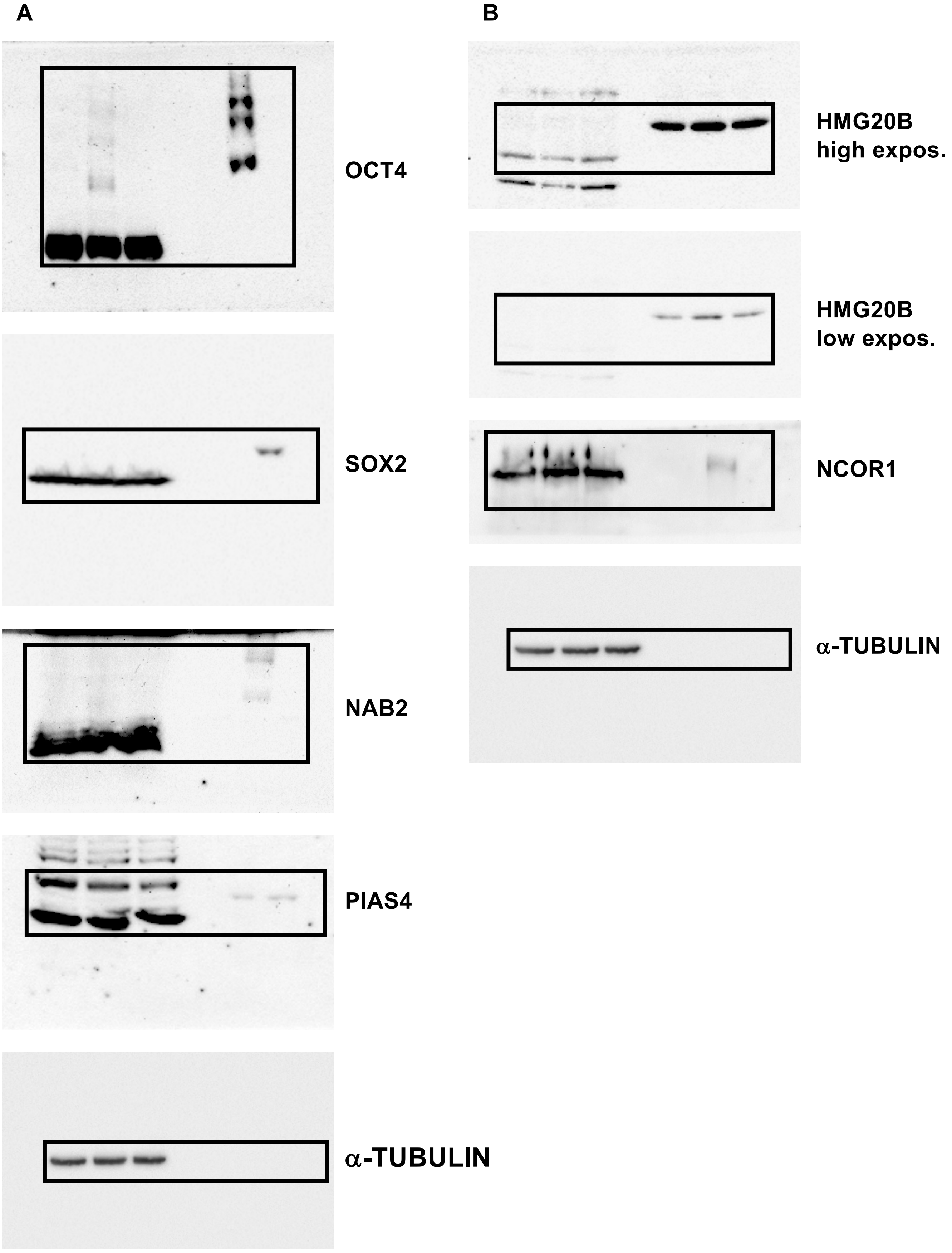

Corresponds to Fig. 4

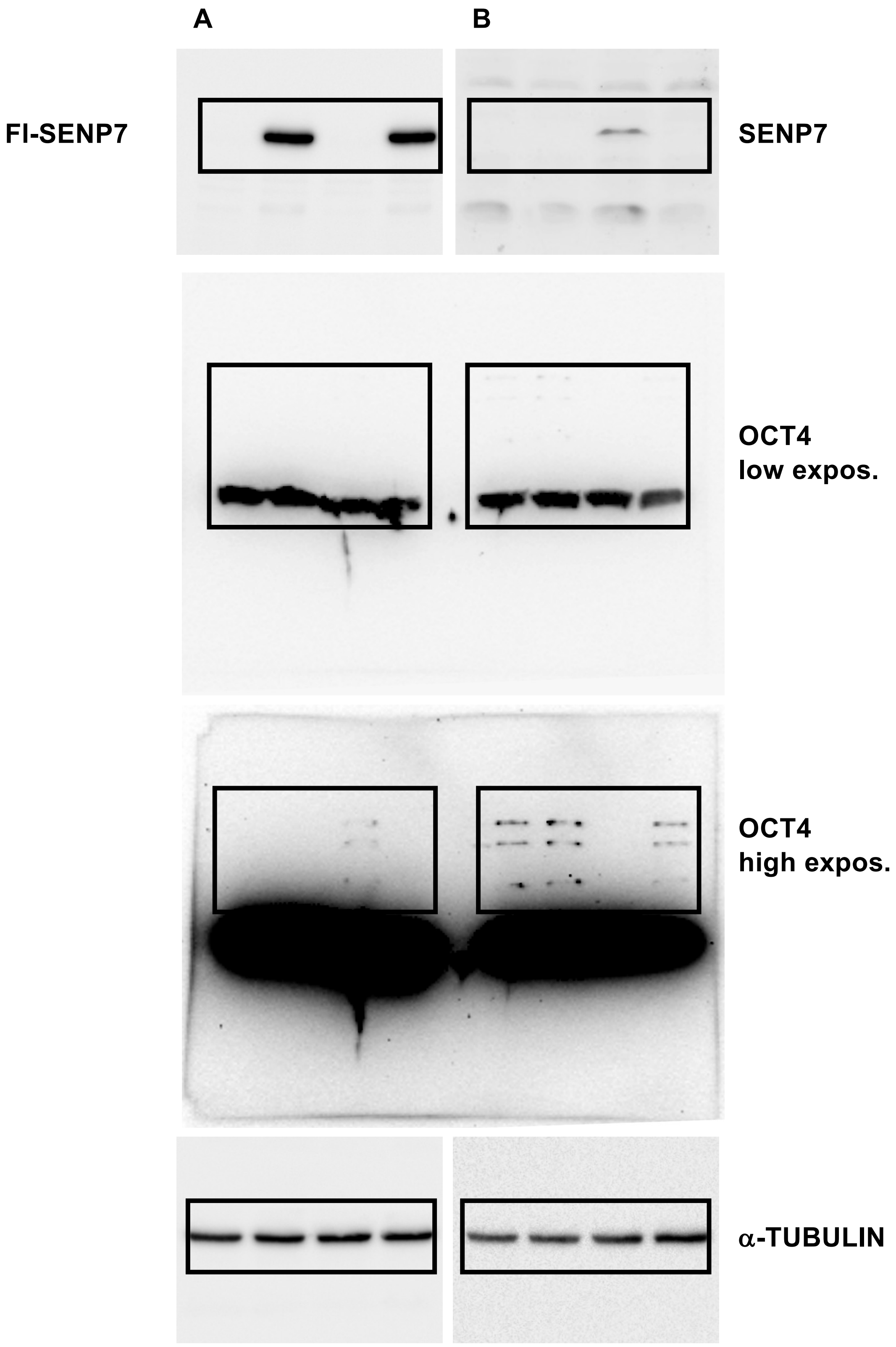

Corresponds to Fig. 5

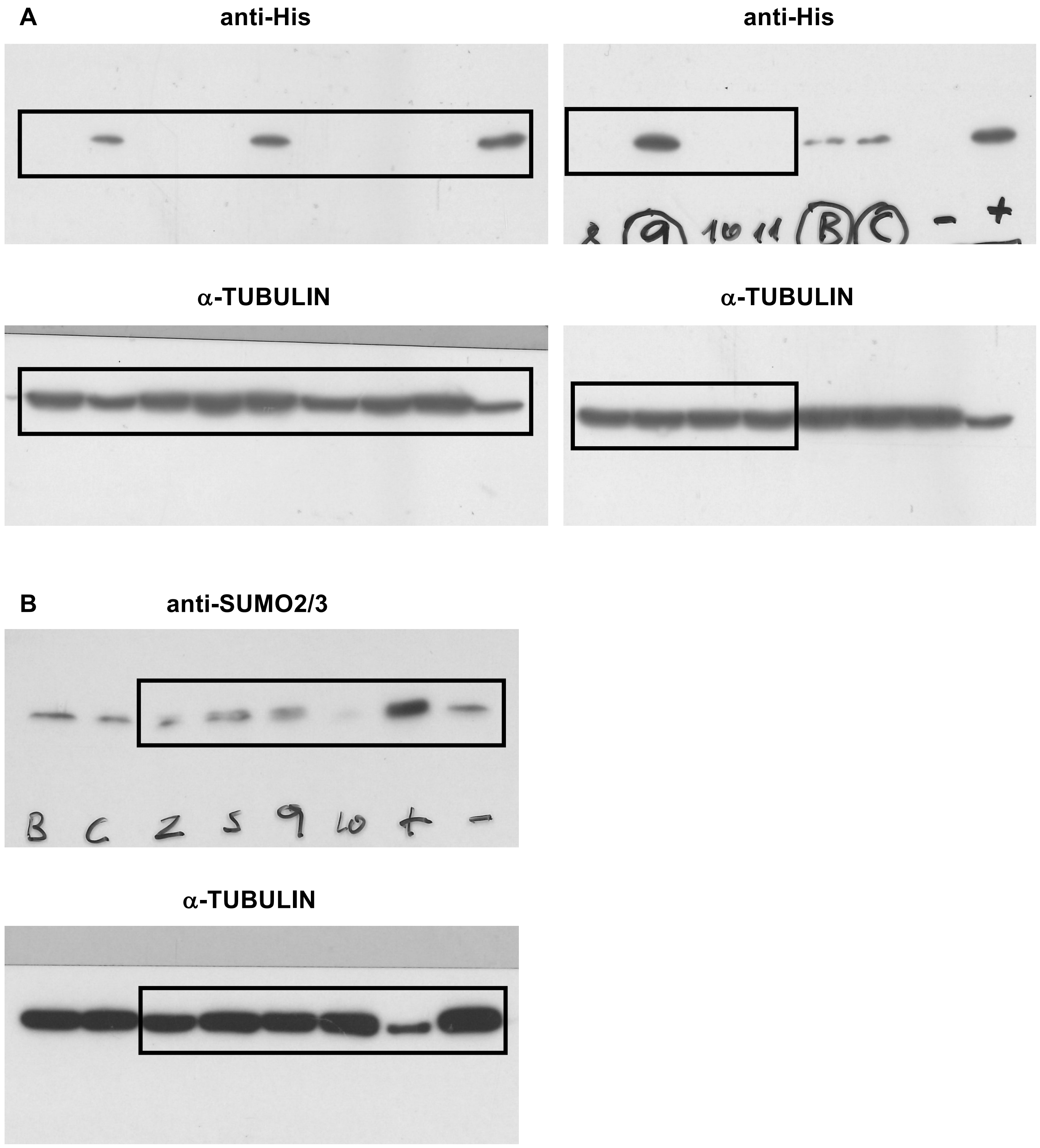

Corresponds to Supplementary Fig. S1

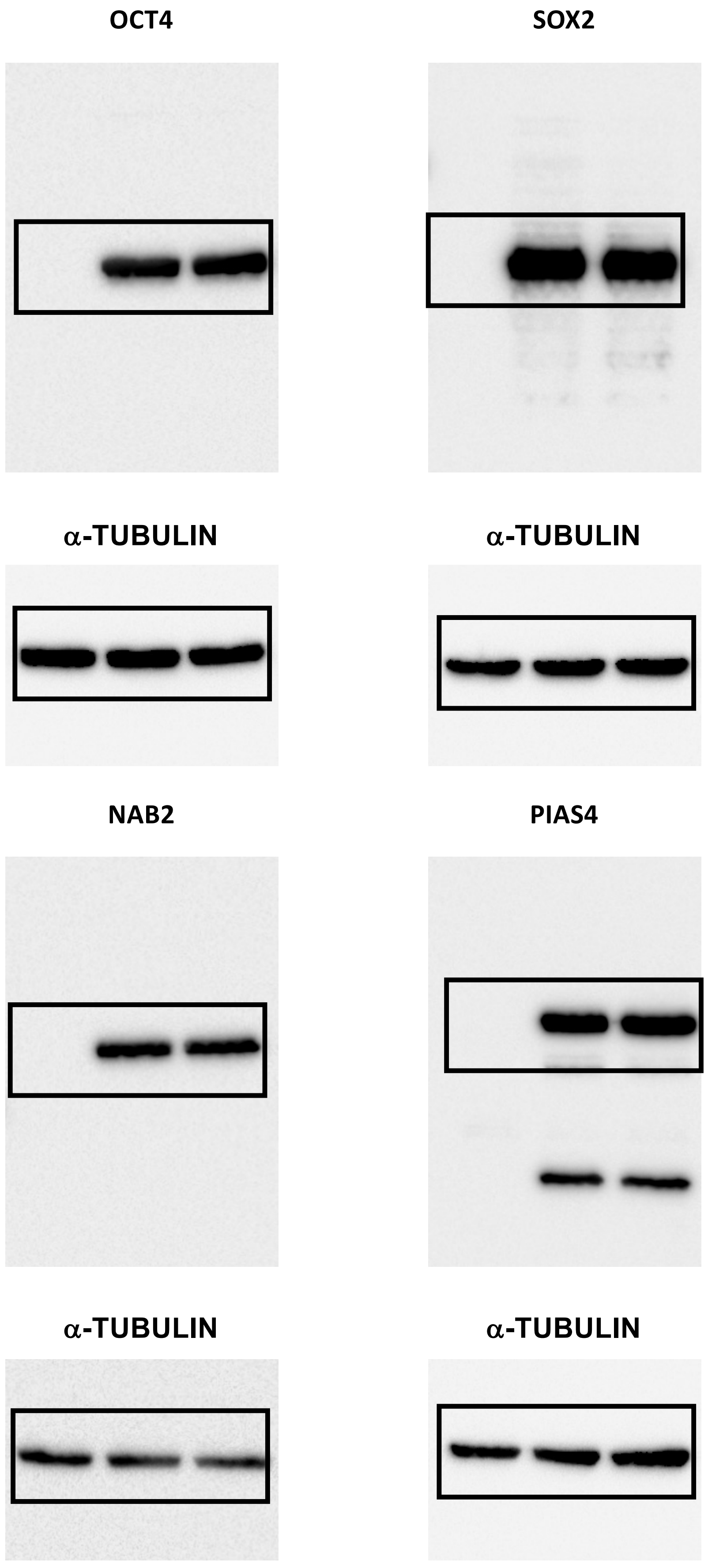

Corresponds to Supplementary Fig. S4B
